# Neural circuit dynamics of drug-context associative learning in the mouse hippocampus

**DOI:** 10.1038/s41467-022-34114-x

**Published:** 2022-11-07

**Authors:** Yanjun Sun, Lisa M. Giocomo

**Affiliations:** Department of Neurobiology, Stanford University School of Medicine, Stanford, CA 94305 USA

**Keywords:** Hippocampus, Neural circuits

## Abstract

The environmental context associated with previous drug consumption is a potent trigger for drug relapse. However, the mechanism by which neural representations of context are modified to incorporate information associated with drugs of abuse remains unknown. Using longitudinal calcium imaging in freely behaving mice, we find that unlike the associative learning of natural reward, drug-context associations for psychostimulants and opioids are encoded in a specific subset of hippocampal neurons. After drug conditioning, these neurons weakened their spatial coding for the non-drug paired context, resulting in an orthogonal representation for the drug versus non-drug context that was predictive of drug-seeking behavior. Furthermore, these neurons were selected based on drug-spatial experience and were exclusively tuned to animals’ allocentric position. Together, this work reveals how drugs of abuse alter the hippocampal circuit to encode drug-context associations and points to the possibility of targeting drug-associated memory in the hippocampus.

## Abstract (web summary)

Drug-associated contexts are a strong trigger for relapse to substance use. Here, the authors report that a subpopulation of neurons in the hippocampus of mice specifically encode drug-associated contextual information.

## Introduction

A core challenge of long-term recovery from drug addiction is the associated high relapse rate, in which a person returns to drug use after a period of abstinence^1^. One of the strongest triggers for relapse in both humans and animal models is re-exposure to a drug-associated environmental context^2–6^. During repeated drug use, a given environmental context is passively associated with the rewarding effects of the drug, such that a previously neutral context may become a conditioned stimulus that can reliably reinstate drug-seeking behavior^7,8^. While mesolimbic dopamine broadcasts a general reward signal that likely supports this process, converging evidence also suggests that reward associative learning relies on multiple memory systems, including the nucleus accumbens (NAc), amygdala, and hippocampus, working in parallel to integrate the necessary sensorimotor information^5,9–15^. In particular, the hippocampus may play a critical role in drug associative learning and the reinstatement of drug seeking behavior, by providing a neural representation of the spatial or contextual information associated with previous drug use^16–23^.

The hippocampus contains place cells that fire in one or few restricted spatial locations in a given environment^24,25^. As a population, place cells construct a map-like representation for the environmental space^25,26^. Across different spatial environments, or contexts, place cells can show uncorrelated activity, with place fields turning on, off, or firing in a new spatial position. These changes in place field activity are collectively referred to as place cell ‘remapping’, which encompasses two phenomena: a change in the firing rate of a place field (rate remapping) and a change in the spatial location of the place field (global remapping)^26–39^. Importantly, place cell representations also track the presence of reward, such as food or water, by clustering their place fields near reward-associated locations, resulting in an over-representation of reward locations^36,40–46^. These observations of place cell remapping have lent significant support to the theory that the hippocampus contains the neural representations needed to encode the combination of sensory cues and internal states that define a given spatial context^38,47^. However, it is unknown whether and how hippocampal place cell representations remap in a maladaptive manner to encode or maintain drug-context associations.

As drug-context associations require repeated drug exposures over an extended period of time, following the activity of the same individual neurons across days is critical for investigating how neurons change their firing patterns over the course of drug-context learning. Here, we used miniscopes to image calcium activity in freely moving mice^48–50^ to examine the representation change of hippocampal place cells over the course of conditioned place preference (CPP)^51,52^. We identified a subset of CA1 place cells that switched off their activity in the saline-paired context after drug conditioning. This form of remapping in a subset of place cells generalized across addictive drugs (methamphetamine, morphine) but was not seen under natural reward conditions. By focusing on the effects of methamphetamine (MA), a psychostimulant that alters catecholaminergic signaling^53^, we found this subset of place cells showed orthogonal activity patterns between the two CPP contexts after drug conditioning, with the resulting remapped pattern predictive of CPP behavior. Our work reveals a potential mechanism in the hippocampus for encoding associations between spatial contexts and addictive drugs via a sub-population of hippocampal neurons.

## Results

### Imaging of CA1 cells in a conditioned place preference (CPP) paradigm

**Fig. 1:**
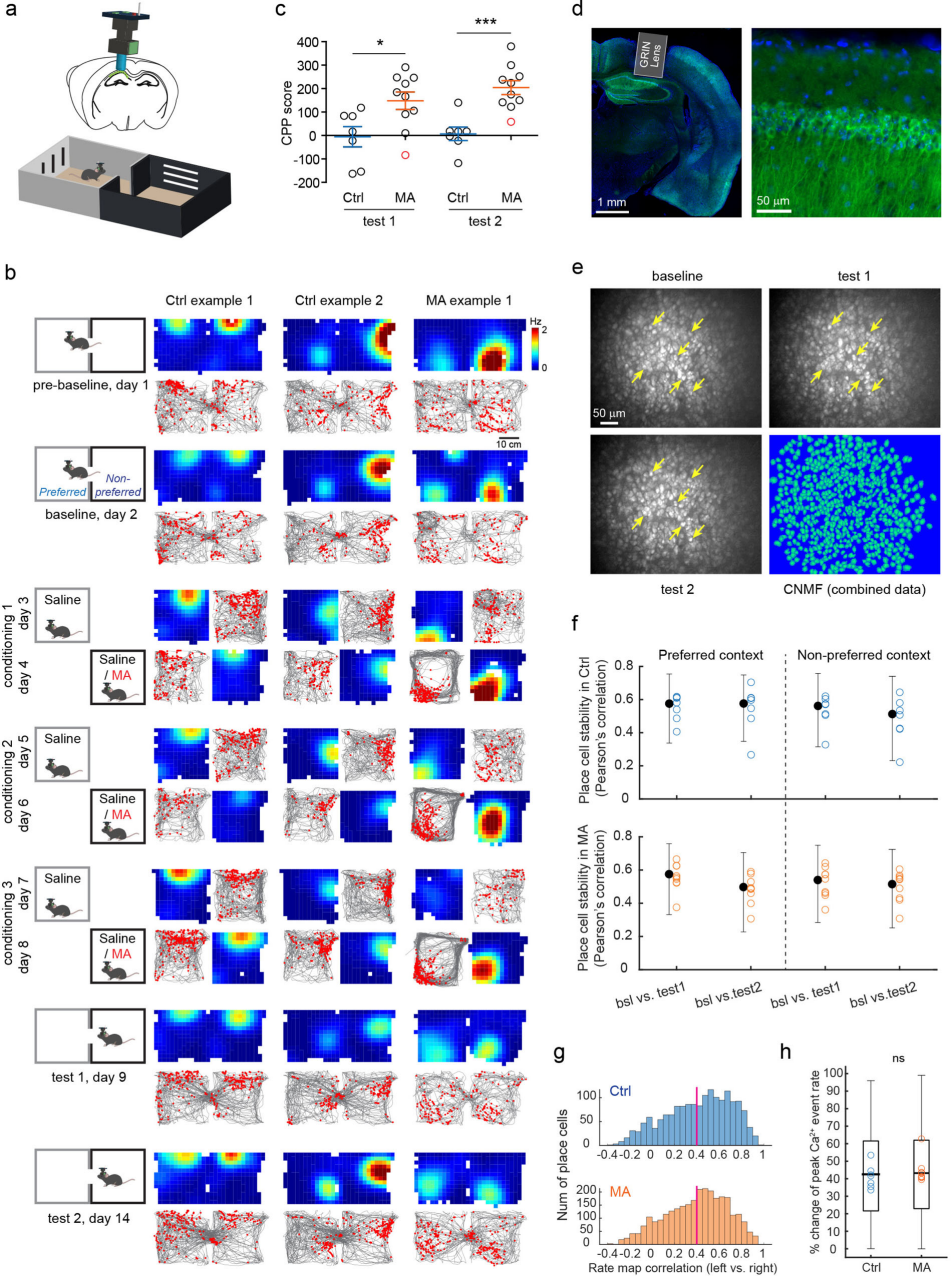
Imaging of CA1 cells in a conditioned place preference (CPP) paradigm. **a** Schematic of calcium imaging using a miniscope and illustration of the CPP box. **b** Schematic of the CPP design and corresponding place cell examples from Ctrl and MA mice. Each column is a cell with activity tracked across all the sessions. Both firing rate map (warmer colors indicate higher firing rates) and raster plot (red dots) on top of the animal’s running trajectory (gray traces) are shown. **c** CPP scores for Ctrl and MA mice (* t(15) = 2.68, *p* = 0.017; *** t(15) = 4.59, *p* = 0.0004, *n* = 7 and 10, respectively, two-tailed unpaired *t*-test). The red data point was excluded from further analysis due to a neutral effect to MA (i.e., a negative CPP score). **d** Histology of GRIN lens implantation. Green: GCaMP6, Blue: DAPI. Right, enlarged view of GCaMP6-expressing CA1 pyramidal cells. Experiment replicated in 29 mice with similar results. **e** Maximum intensity projections of CA1 imaging for indicated sessions from a representative mouse. Yellow arrows point to representative tracked neurons. Bottom right, CNMF-E spatial footprints of neurons. Characterization replicated in 54 mice, see Supplementary Fig. 1. **f** Long-term stability (Pearson’s correlation) in Ctrl (blue) and MA (orange) mice. Black bars show median and interquartile range for all place cells from all mice (*n* = 1680 and 2882 cells, respectively), circles indicate median for each mouse (*n* = 7 and 9). bsl: baseline. **g** Inter-compartment rate map correlations of place cells in the baseline for Ctrl (median: 0.46, *n* = 1680 cells from 7 mice) and MA mice (median: 0.43, *n* = 2882 cells from 9 mice). The red line (=0.4) separates rate vs. non-rate remapping place cells. **h** Percentage change in peak Ca^2+^ event rates (peakER) for rate remapping place cells across the two contexts in baseline. For each place cell, the value is defined as abs(peakER_left_−peakER_right_)/max(peakER_left_, peakER_right_). Box plots show values for all the rate remapping place cells from all mice (median: Ctrl = 42.5%, MA = 43.2%, *Z* = −0.06, *p* = 0.95, *n* = 932 and 1556 cells, respectively, two-tailed rank-sum test), while circles indicate median for each mouse. For each box plot, the center indicates median, the box indicates 25th and 75th percentiles. The whiskers extend to the most extreme data points without outliers. ns: not significant.

To examine the effects of drug-context associations on the activity of CA1 neurons, we performed in vivo single photon (1P) miniscope calcium imaging in mice during a conditioned place preference (CPP) paradigm (Fig. 1). The CPP apparatus consisted of two compartments, with distinct contexts defined by different colors and visual cues (Fig. 1a, b). After two days of habituation, mice were allowed access to both CPP contexts for the pre-baseline and baseline sessions (day 1 = pre-baseline; day 2 = baseline) (Fig. 1b). An animal’s natural context preference was determined on day 2 (baseline session) (Fig. 1b). For conditioning (*n* = 3 sessions), saline injections were paired with the naturally preferred context, while methamphetamine (MA) injections were paired with the naturally non-preferred context (Fig. 1b). One and six days after the conditioning, two test sessions (test 1 and test 2, respectively) were performed to assess post-conditioning place preference (Fig. 1b). Control (Ctrl) mice (*n* = 7 mice) underwent the same protocol but saline injections were paired with both contexts during the conditioning (Fig. 1b). In MA mice (*n* = 10 mice), we observed significant place preference for the MA-paired context in the test sessions (mean ± SEM, test 1: Ctrl vs. MA, −5.62 ± 43.28 vs. 147.80 ± 37.21 s; test 2: Ctrl vs. MA, 6.89 ± 28.67 vs. 204.10 ± 29.74 s, *n* = 7 and 10, respectively; Fig. 1c, “Methods”).

We used Ai94; Camk2a-tTA; Camk2a-Cre transgenic mice (Fig. 1d) to enable stable GCaMP6s expression in CA1 pyramidal neurons and single cell tracking across multiple days, as demonstrated in the maximum projected images and the colocalization analysis from different imaging sessions after alignment (Fig. 1e, Supplementary Fig. 1a–d, Supplementary Movies 1–3). We extracted calcium signals, using a CNMF-based method^54^, from aligned and temporally concatenated image data for each animal. This method has shown improved cell registration from calcium signals^48,55^ (Fig. 1e, Supplementary Fig. 1a). Calcium signals were then binarized into deconvolved spikes^56^, which we treated as calcium events (Supplementary Fig. 1e).

In both Ctrl and MA animals, we observed stable spatial representations of CA1 place cells in both CPP contexts across days (median correlation, Ctrl: preferred context, baseline (bsl) vs. test 1 = 0.57, bsl vs. test 2 = 0.58; non-preferred context, bsl vs. test 1 = 0.56, bsl vs. test 2 = 0.51; *n* = 1680 place cells from 7 mice; MA: preferred context, bsl vs. test 1 = 0.58, bsl vs. test 2 = 0.50; non-preferred context, bsl vs. test 1 = 0.54, bsl vs. test 2 = 0.52; *n* = 2882 place cells from 9 mice), with place cells defined by their activity in the baseline session (Fig. 1b, f). Across the two CPP contexts within the baseline session, we observed signatures of both rate and global remapping for place cells (Fig. 1b). For both Ctrl and MA mice, place cells showed similar inter-compartment (between the left and right CPP contexts) spatial correlations in the baseline session, with ~55% place cells showing signatures of rate remapping (spatial correlation > 0.4) (Fig. 1g). For rate remapped place cells, we observed a ~43% change in peak calcium event rates between place fields across the two contexts (Fig. 1h). Previous work has reported that when animals are exposed to different novel environments, place cell representations become increasingly orthogonal between the environments as a function of experience^37^. However, in Ctrl mice, inter-compartment spatial correlations of place cells remained constant over baseline and test session (median correlation: 0.43, 0.49, and 0.46, for baseline, test 1, and test 2 in Ctrl mice, respectively), suggesting the observed rate remapping in the baseline session is not due to insufficient experience in the CPP environment. Together these data demonstrate that in baseline sessions, CA1 place cells in both Ctrl and MA mice remap between the two contexts of a CPP paradigm.

### MA-conditioning results in a context-specific decrease in place cell number

**Fig. 2:**
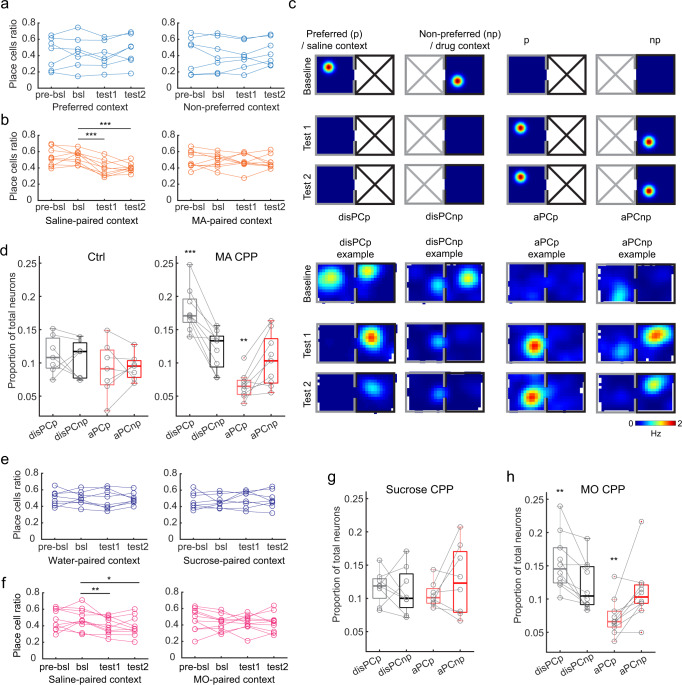
Drug-conditioning results in an unbalanced rate of place cell turnover across the two CPP contexts. **a** The ratio of place cells over the total number of neurons in Ctrl mice in the preferred (F(3, 6) = 1.21, *p* = 0.34, *n* = 7 mice, repeated measures ANOVA) and non-preferred context (F(3,6) = 0.85, *p* = 0.49), respectively. pre-bsl: pre-baseline, bsl: baseline. **b** Same as (**a**) but for MA mice (saline-paired context: F(3,8) = 16.36, *p* < 0.0001; MA-paired context: F(3,8) = 0.31, *p* = 0.82, *n* = 9 mice, repeated measures ANOVA; *** bsl vs. test 1: t(8) = 6.30, *p* = 2.34 × 10^−4^; bsl vs. test 2: t(8) = 5.90, *p* = 3.64 × 10^−4^, *n* = 9 mice, two-tailed paired *t*-test). **c** Top: schematic of disPCp, disPCnp, aPCp, and aPCnp. These functional cell types were defined by their activity change in either context of the CPP apparatus. Gaussian spots represent place fields with warm colors indicating a high firing rate. Bottom: rate maps of representative cells for each of the functional cell types. **d** Proportions of functional cell types in Ctrl (disPCp vs. disPCnp: t(6) = 0.57, *p* = 0.59; aPCp vs. aPCnp: t(6) = 0.23, *p* = 0.83, *n* = 7 mice) and MA mice (*** disPCp vs. disPCnp: t(8) = 6.16, *p* = 2.7 × 10^−4^; ** aPCp vs. aPCnp: t(8) = −3.66, *p* = 0.006, *n* = 9 mice, two-tailed paired *t*-test). **e** Same as (**a**) but for sucrose mice (water-paired context: F(3,7) = 0.084, *p* = 0.97; sucrose-paired context: F(3,7) = 0.46, *p* = 0.71, *n* = 8 mice, repeated measures ANOVA). **f** Same as (**a**) but for morphine (MO) mice (saline-paired context: F(3,9) = 6.56, *p* = 0.0018; MO-paired context: F(3,9) = 0.62, *p* = 0.61, *n* = 10 mice, repeated measures ANOVA; ** bsl vs. test 1: t(9) = 3.5, *p* = 0.0067; * bsl vs. test 2: t(9) = 2.50, *p* = 0.034, *n* = 10 mice, two-tailed paired *t*-test). **g** Same as (**d**) but for sucrose mice (disPCp vs. disPCnp: t(7) = 0.44, *p* = 0.67; aPCp vs. aPCnp: t(7) = −1.28, *p* = 0.24, *n* = 7 mice, two-tailed paired *t*-test). **h** Same as (**d**) but for MO mice (** disPCp vs. disPCnp: t(9) = 3.78, *p* = 0.004; aPCp vs. aPCnp: t(9) = −3.32, *p* = 0.0089, *n* = 10 mice, two-tailed paired *t*-test). For box plots throughout the figure, the center indicates median, the box indicates 25th and 75th percentiles. The whiskers extend to the most extreme data points without outliers. Circles denote data points for each mouse. For all figure panels, *p < 0.05, **p < 0.01, ***p < 0.001.

We next considered whether MA-conditioning induced changes in CA1 place cells at the population level. We calculated the proportion of place cells over time for each animal in a context-specific manner. In Ctrl mice, the number of place cells remained the same across sessions in both contexts (Fig. 2a). However, in MA mice, we found a significant decrease in the number of place cells specifically in the saline-paired context in the test sessions compared to baseline (mean ± SEM, baseline: 0.53 ± 0.03, test 1: 0.39 ± 0.03, test 2: 0.40 ± 0.02, *n* = 9; Fig. 2b). As the activity of place cells changes over time^49^, this decrease in place cell number in MA mice could either reflect the loss of existing place cells or the absence of the addition of new place cells. To examine these possibilities, we defined four functional cell types based on how their context-specific activity changed between baseline and test sessions (Fig. 2c). Namely, cells with place fields in the preferred context in baseline that lost their spatial tuning in the same context in both test sessions were defined as disPCp (disappeared place cells in the preferred context; note the preferred context is equivalent to the saline-paired context in MA mice). A similar definition was applied to disPCnp for the non-preferred context (disappeared place cells in the non-preferred context; note the non-preferred context is equivalent to the MA-paired context in MA mice). Cells with no spatial tuning in baseline but a place field in the preferred or non-preferred context for both test sessions were defined as aPCp (appeared place cells in the preferred context) and aPCnp (appeared place cells in the non-preferred context), respectively (Fig. 2c). Quantifying the proportion of these four cell types revealed that Ctrl mice showed a stable turnover rate of place cells across the two contexts (mean ± SEM, disPCp vs. disPCnp: 0.11 ± 0.01 vs. 0.11 ± 0.01; aPCp vs. aPCnp: 0.09 ± 0.01 vs. 0.09 ± 0.01, *n* = 7; Fig. 2d). However, in MA mice, the proportion of disPCp was greater than disPCnp, while the proportion of aPCp was lower than aPCnp (mean ± SEM, disPCp vs. disPCnp: 0.18 ± 0.01 vs. 0.12 ± 0.01; aPCp vs. aPCnp: 0.066 ± 0.007 vs. 0.10 ± 0.01, *n* = 9; Fig. 2d). This result indicates that, in MA mice, more place cells disappeared and fewer place cells appeared in the originally preferred (saline-paired) context compared to the non-preferred (MA-paired) context. This effect did not result from a change in the animals’ spatial coverage across the two CPP contexts, or a running speed and head direction sampling difference between baseline and test sessions (Supplementary Fig. 2a–f). These results thus suggest that MA conditioning induced an overall loss of place fields specifically in the saline-paired context.

### Context-specific loss of place cells was specific to drug rewards

To compare the MA-induced place cell changes to what occurs during natural reward learning, we performed sucrose conditioned place preference (Sucrose CPP) while imaging hippocampal activity in a separate cohort of mice (*n* = 8 mice) (Supplementary Fig. 3a). Pairing sucrose with the naturally non-preferred context induced a significant place preference shift in test sessions (Supplementary Fig. 3b) without affecting the animals’ speed and head direction sampling (Supplementary Fig. 2g). The degree of place preference shift in sucrose mice was comparable to that observed in MA mice (Supplementary Fig. 3c). However, for sucrose mice, the number of place cells across sessions remained stable in both contexts and the rate of place cell turnover did not differ between the two contexts (mean ± SEM, disPCp vs. disPCnp: 0.12 ± 0.009 vs. 0.11 ± 0.01; aPCp vs. aPCnp: 0.11 ± 0.007 vs. 0.13 ± 0.02, *n* = 8; Fig. 2e, g).

We next considered whether the changes we observed in place cells in MA mice occurred in response to other addictive drugs. In a separate cohort of mice, we performed morphine conditioned place preference (MO CPP) while imaging hippocampal activity (*n* = 12 mice) (Supplementary Fig. 3d, e). In MO mice, between baseline and test sessions, we observed a decrease in the number of place cells for the saline-paired context (mean ± SEM, baseline: 0.48 ± 0.04, test 1: 0.40 ± 0.03, test 2: 0.39 ± 0.04, *n* = 10) as well as an unbalanced rate of place cell turnover between the two contexts (mean ± SEM, disPCp vs. disPCnp: 0.15 ± 0.01 vs. 0.12 ± 0.01; aPCp vs. aPCnp: 0.073 ± 0.009 vs. 0.11 ± 0.01, *n* = 10; Fig. 2f, h). These place cell changes were consistent with those observed in MA mice. In addition, experiments using a conditioned place aversion protocol showed a distinct effect on place cells compared to the CPP experiments, supporting the idea that drug-induced place cell changes reflect the rewarding effects of the drug, rather than drug withdrawal, a preference shift or the stereotyped behavior observed during conditioning sessions (Supplementary Figs. 2h–j, 3f–j). Together, these results indicate that the changes in place cell activity observed in drug CPP are due to an association between the rewarding effects of the drug and the corresponding spatial context.

### The activity of disPCp encodes the drug-context association

To further consider the relationship between changes in place cell activity and drug-context associations, we focused on MA CPP. First, to examine whether the unbalanced rate of place cell turnover impacts the accuracy of spatial coding, we trained a Naive Bayes classifier using baseline data and examined the accuracy of position decoding using test session data (Supplementary Fig. 4a, “Methods”). We found that despite the context-specific loss of place cells in MA mice, Ctrl and MA mice showed comparable overall decoding performance, and MA mice showed comparable decoding accuracy between the preferred and non-preferred contexts (Supplementary Fig. 4b–h).

**Fig. 3:**
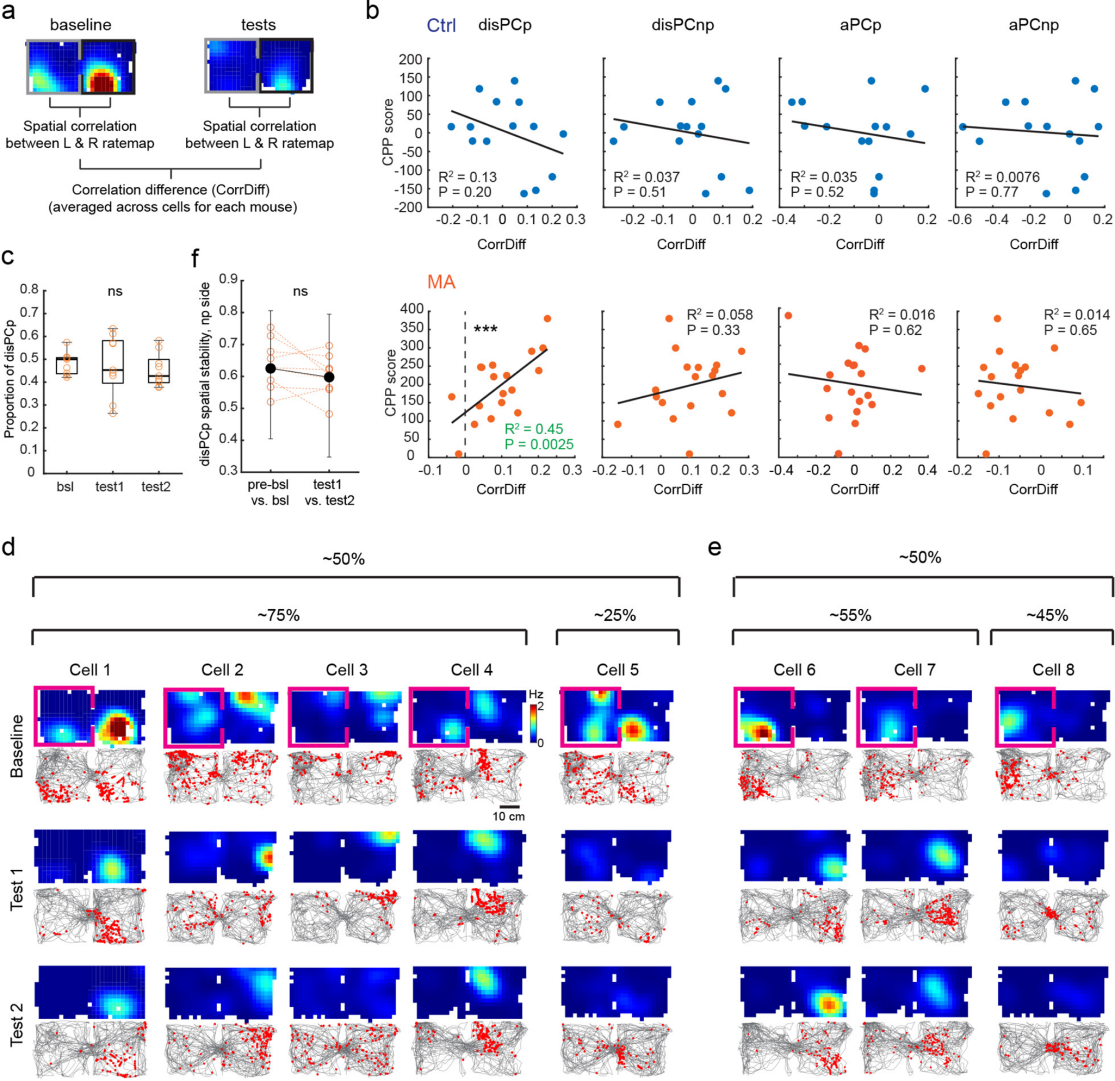
The activity of disPCp encodes the drug-context association. **a** Schematic for calculating inter-compartment correlation difference (CorrDiff) between baseline and test (test 1 or test 2) sessions for each neuron. **b** CorrDiff calculated using disPCp, but not other cell types, was significantly correlated with behavioral CPP scores in MA mice (orange, bottom). This correlation was not seen in Ctrl mice (blue, top). Each dot on the x-axis is a CorrDiff value for a mouse calculated using the designated cell types indicated on top. Each mouse has two CorrDiff values (baseline vs. test 1 and baseline vs. test 2). CorrDiff of disPCp in MA mice was also significantly higher than zero (*Z* = 3.55, *p* = 3.86 × 10^−4^, *n* = 18 session pairs, two-tailed sign-rank test against zero). **c** The proportion of disPCp showing significant spatial tuning in the MA-paired context in MA mice (mean ± SEM, bsl: 0.48 ± 0.02; test 1: 0.47 ± 0.04; test 2: 0.45 ± 0.02; F(2,8) = 0.25, *p* = 0.78, *n* = 9 mice, repeated measures ANOVA). bsl: baseline. For box plot, the center indicates median, the box indicates 25th and 75th percentiles. The whiskers extend to the most extreme data points without outliers. Circles denote data points for each mouse. ns, not significant. **d**, **e** Categories of disPCp in MA mice with single neuron examples. Each column is a cell with activity tracked from baseline to test sessions. Top plots are rate maps with warmer colors indicating higher firing rates. Bottom plots show the mouse’s trajectory (gray) and detected calcium events (red). Pink box indicates the session and context in which the place cell was defined. Plots are aligned such that the saline-paired context (naturally preferred context) is on the left. **f** Spatial stability (Pearson’s correlation) of disPCp in the MA-paired context in MA mice (*p* = 0.20, *n* = 9 mice, two-tailed sign-rank test). Black bars show median and interquartile range of values for all place cells from all mice, while circles indicate median for each mouse.

We next considered whether the activity of the four functional cell types (disPCp, disPCnp, aPCp, aPCnp) correlated with the CPP behavior. We quantified an inter-compartment spatial correlation for baseline and test sessions using each of the four functional cell types (Fig. 3a). Strikingly, the spatial correlation difference of disPCp (CorrDiff, baseline – test; Fig. 3a) was significantly positively correlated with the behavioral CPP score in MA mice (Fig. 3b). This correlation with behavior was not observed in Ctrl mice or for any other functional cell type (Fig. 3b). Interestingly, the CorrDiff value for disPCp was significantly higher than zero in MA mice (Fig. 3b), suggesting that drug conditioning resulted in disPCp exhibiting more uncorrelated inter-compartment representations for the two CPP contexts in test sessions. To further characterize the correlation of disPCp with CPP behavior, we quantified the type of activity change that disPCp exhibited. First, in MA mice, 34 ± 3% of place cells with place fields in the MA-paired context were disPCp. Furthermore, ~50% of disPCp showed significant spatial tuning in the MA-paired context and this percentage remained similar between baseline and test sessions (Fig. 3c). Next, we split disPCp into two groups based on their activity in the baseline session; Group 1 (48 ± 2%, *n* = 9): disPCp with significant place fields in both contexts in baseline (Fig. 3d); Group 2 (52 ± 2%): disPCp with a significant place field only in the preferred (i.e., saline-paired) context in baseline (Fig. 3e). Within Group 1, 76 ± 4% of disPCp maintained their spatial tuning in the MA-paired context in at least one of the test sessions, while the remaining 24 ± 4% lost their spatial tuning (Fig. 3d). Within Group 2, 54 ± 5% of disPCp gained a new place field in the MA-paired context in at least one of the test sessions, while the remaining 46 ± 5% did not gain a new place field (Fig. 3e). These quantifications indicate that the correlation between the CorrDiff value for disPCp and CPP behavior likely reflects the cumulative result of heterogeneous remapping patterns in disPCp. Finally, the spatial stability (as measured within the baseline or test sessions) of MA disPCp in the drug-paired context was not affected by drug-conditioning (Fig. 3f). Together, these data suggest that drug-context associations differentiate the spatial representations of disPCp across the two CPP contexts, resulting in a cumulative remapping pattern predictive of CPP behavior.

### disPCp contribute to the encoding of MA-paired context after conditioning

**Fig. 4:**
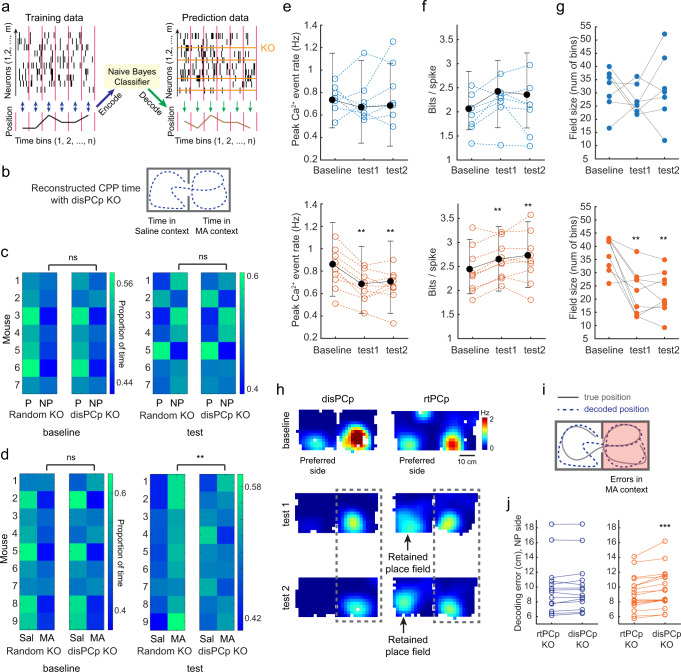
disPCp contribute to the encoding of MA-paired context after conditioning. **a** Schematic of the knock-out (KO) decoding method. **b** Evaluating the decoding performance using reconstructed CPP time. **c** Reconstructed CPP time with disPCp vs. random neuron KO in Ctrl mice. Each row is a mouse and each column represents a CPP context (P: preferred, NP: non-preferred). Brighter colors indicate a larger proportion of time. Left, baseline analyses (*p* = 0.08, *n* = 7 mice, two-tailed sign-rank test). Right, test analyses (*p* = 0.11, *n* = 7 mice, two-tailed sign-rank test). ns, not significant. **d** Organized as in (**c**), for MA mice. Baseline analyses (*p* = 0.2, *n* = 9 mice, two-tailed sign-rank test). Test analyses (*p* = 0.0039, *n* = 9 mice, two-tailed sign-rank test). See results. **e**–**g** Place field properties of disPCp in MA-paired context for Ctrl (blue) and MA (orange) mice. **e** Peak calcium event rate: Ctrl (baseline vs. test 1: *p* = 0.81; baseline vs. test 2: *p* = 0.81, *n* = 7 mice); MA (baseline vs. test 1: *p* = 0.0039; baseline vs. test 2: *p* = 0.0078, *n* = 9 mice, two-tailed sign-rank test). **f** Spatial information: Ctrl (baseline vs. test 1: *p* = 0.11; baseline vs. test 2: *p* = 0.47, *n* = 7 mice); MA (baseline vs. test 1: *p* = 0.0078; baseline vs. test 2: *p* = 0.0039, *n* = 9 mice, two-tailed sign-rank test). Black bars show median and interquartile range for all disPCp from all mice. Circles indicate median for each animal. **g** Field size: Ctrl (baseline vs. test 1: *p* = 0.30; baseline vs. test 2: *p* = 1, *n* = 7 mice); MA (baseline vs. test 1: *p* = 0.0078; baseline vs. test 2: *p* = 0.0039, *n* = 9 mice, two-tailed sign-rank test), **h** An example of disPCp vs. rtPCp. The gray dashed rectangle highlights the drug-paired context. **i** Schematic for calculating decoding errors in the drug-paired context. **j** Decoding errors for Ctrl (blue) and MA (orange) mice by KO rtPCp vs. disPCp (Ctrl: *p* = 0.09, *n* = 14 sessions from 7 mice; MA: Z = −3.59, *p* = 3.27 × 10^−4^, *n* = 18 sessions from 9 mice, two-tailed sign-rank test).

To assess the degree to which disPCp contribute to the encoding of drug-context associations we applied a computational knockout (KO) decoding analysis based on the aforementioned naive Bayes classifier (Fig. 4a, Supplementary Fig. 4, “Methods”). In the KO decoding analyses, we computationally removed either disPCp or a random group of neurons (sample-size matched) from the data set before feeding them into the trained classifier for making predictions (Fig. 4a). We performed two sets of computational KO decoding analyses to investigate the contribution of disPCp to encoding the CPP contexts before and after drug conditioning. First, we trained the decoder on one of the baseline sessions and then used the decoder to make the predictions on the other baseline session. Second, we trained the decoder on one of the test sessions and then used the decoder to make predictions regarding the time spent in each CPP compartment on the other test session. To directly visualize the contribution of disPCp to CPP behavior (i.e., the time spent in each CPP compartment), we plotted the reconstructed CPP time generated by the decoder for each individual mouse as heatmaps for each CPP context (Fig. 4b–d). In Ctrl mice, there was no significant difference in the reconstructed CPP time for random versus disPCp KO in either the baseline (proportion of total time in the non-preferred context, mean ± SEM, disPCp KO: 0.47 ± 0.01; random KO: 0.47 ± 0.01, *n* = 7) or the test analysis (mean ± SEM, disPCp KO: 0.50 ± 0.02; random KO: 0.51 ± 0.02, *n* = 7; Fig. 4c). Note, given the high decoding accuracy in both contexts (Supplementary Fig. 4), the results from the random KO condition largely recapitulated the true behavioral CPP time (Supplementary Fig. 4i), with more reconstructed time shown in the naturally preferred context in baseline (left column of Fig. 4c, d). In MA mice, there was no significant difference in the reconstructed CPP time for random versus disPCp KO in the baseline analysis (proportion of total time in the MA context, mean ± SEM, disPCp KO: 0.46 ± 0.02; random KO: 0.44 ± 0.02, *n* = 9; Fig. 4d, left). Notably, however, in the test analysis, the reconstructed CPP time captured the MA-induced place preference for the random KO, but this place preference was disrupted for the disPCp KO (mean ± SEM, disPCp KO: 0.50 ± 0.01; random KO: 0.55 ± 0.01, *n* = 9; Fig. 4d, right). This result was not due to the larger proportion of disPCp in MA compared to Ctrl mice, as matching the proportion of disPCp between MA and Ctrl mice by down-sampling replicated this result (Supplementary Fig. 4j). Together, these results suggest that disPCp in MA mice biased their encoding towards representing the drug-paired context after drug conditioning.

The down-sampling result (Supplementary Fig. 4j) raises the possibility that other factors may contribute to the biased encoding of disPCp, besides the loss of spatial tuning in the saline-paired context. Indeed, the biased encoding of the drug-paired context by disPCp was also reflected in the place cell metrics for disPCp in the MA-paired context. In MA mice, disPCp place fields in the MA-paired context had a decreased peak calcium event rate, increased spatial information and a smaller field size between the baseline and test sessions (Fig. 4e–g, orange plots). These effects were not seen in Ctrl mice (Fig. 4e–g, blue plots). These results suggest that in addition to losing their spatial tuning in the saline-paired context after MA conditioning, disPCp may contribute to the encoding of drug-context associations by sharpening their place fields in the MA-paired context.

We next considered how much disPCp contributed to the encoding of drug-context associations compared to other functionally defined place cell types. We identified a fifth functional cell type that retained spatial tuning in the saline-paired context for at least one test session (termed rtPCp: retained place cells on the preferred side. Note disPCp and rtPCp are mutually exclusive; Fig. 4h). We then compared the contribution of disPCp versus rtPCp to the spatial coding of MA-paired context after conditioning (Fig. 4h, i). We again trained the naive Bayes decoder using data from the baseline session and examined the accuracy of position decoding in the MA-paired context using data from the test sessions (Fig. 4i). In Ctrl mice, rtPCp and disPCp KO showed comparable decoding errors (mean ± SEM: rtPCp KO, 9.95 ± 0.95 cm; disPCp KO, 10.09 ± 0.94 cm, *n* = 14; Fig. 4j). However, in MA mice, disPCp KO resulted in significantly larger decoding errors than rtPCp KO, suggesting that disPCp contributed more to the spatial coding of the MA-paired context than rtPCp in test sessions (mean ± SEM: rtPCp KO, 9.12 ± 0.54 cm; disPCp KO, 9.72 ± 0.62 cm, *n* = 18; Fig. 4j). Together, compared to place cells similarly defined by their activity in the saline-paired context in baseline (rtPCp), disPCp contributed significantly more to the encoding of the drug-associated context after drug conditioning.

### disPCp in MA mice emerge in an experience-dependent manner

**Fig. 5:**
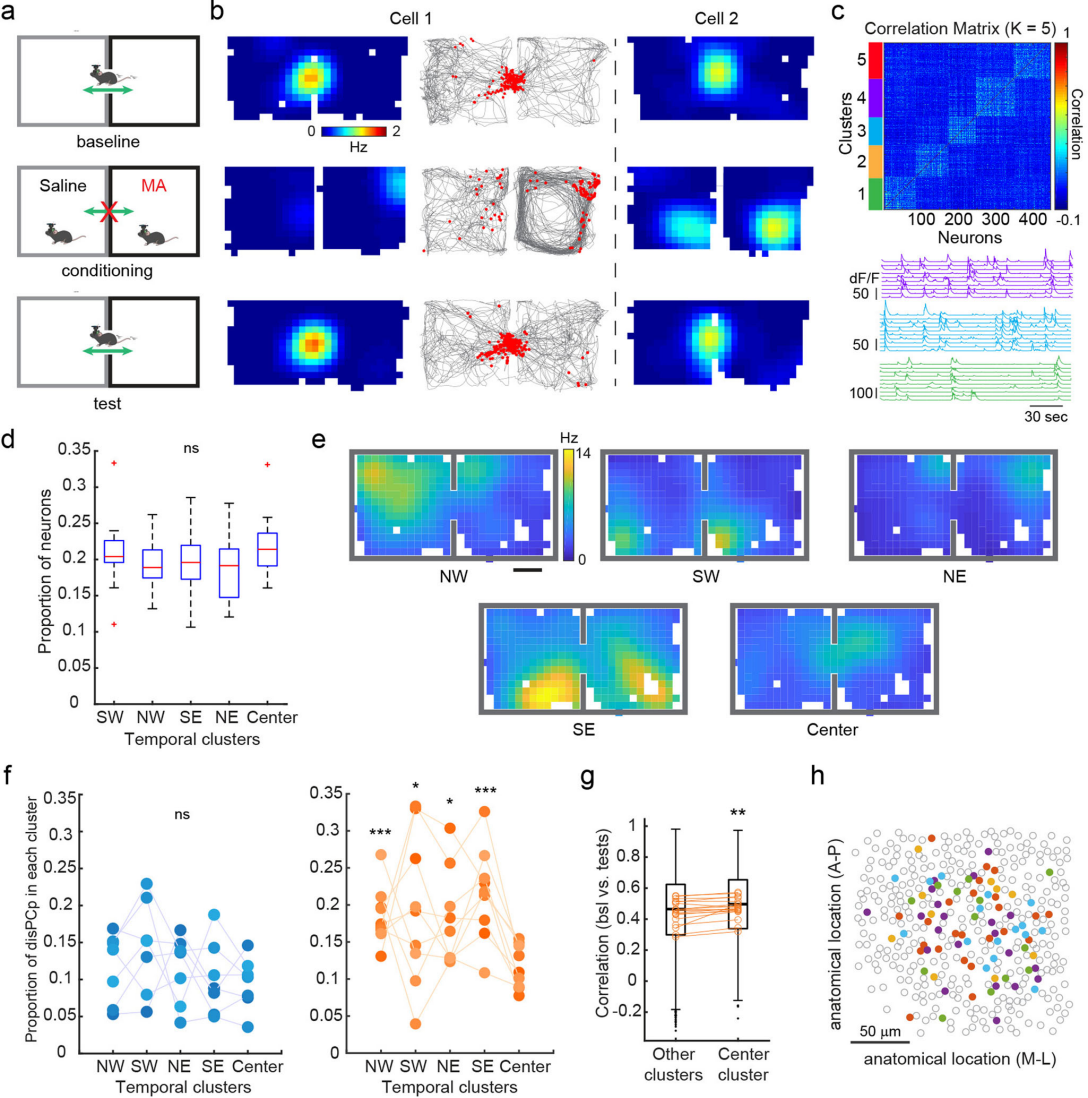
disPCp in MA mice emerge in an experience-dependent manner. **a** Schematic illustrating an animal’s possible location in baseline, conditioning, and test sessions. **b** Two example neurons that are maximally active at the CPP junction. Sessions organized as labeled in (**a**). Both rate map (warmer colors indicate higher firing rates) and raster plot (red calcium events on top of gray running trajectory) are shown for cell 1. **c** Top, the temporal correlation matrix of calcium signals from baseline of an example mouse, sorted by k-means derived temporal clusters. Bottom, synchronized calcium activity for representative neurons from three different clusters (colors correspond to the top panel). **d** The proportion of neurons assigned to each cluster (F(4,75) = 1.77, *p* = 0.14, one-way ANOVA, *n* = 16 mice from Ctrl and MA groups). Box plot: center indicates median, box indicates 25th and 75th percentiles, whiskers extend to the most extreme data points without outliers. Outliers are shown in red plus symbol. SW, southwest; NW, northwest; SE, southeast; NE, northeast. ns, not significant. **e** Summed ensemble rate maps of temporally clustered disPCp from an MA mouse. **f** disPCp showed a non-random distribution across temporal clusters in MA (orange) but not Ctrl (blue) mice (F(4,30) = 1.06, *p* = 0.39 for Ctrl; F(4,40) = 3.01, *p* = 0.029 for MA, one-way ANOVA, *n* = 7 and 9, respectively). In MA mice, the proportion of disPCp in the center cluster was significantly lower compared to other clusters (NW vs. center, t(8) = 5.87, *p* = 1.84 × 10^−4^; SW vs. center, t(8) = 1.95, *p* = 0.043; NE vs. center, t(8) = 2.35, *p* = 0.023; SE vs. center, t(8) = 5.33, *p* = 3.50 × 10^−4^, *n* = 9 mice, one-tailed paired *t*-test). **g** Rate map correlation (Pearson’s) between baseline and test sessions for neurons in center vs. other clusters in MA mice. The box plots show values from all neurons in center (*n* = 740 cells) vs. other clusters (*n* = 3143 cells). Box plot: center bar indicates median, box indicates 25th and 75th percentiles, whiskers extend to the most extreme data points without outliers. Black dots are outliers. Circles indicate mean for each session comparison (**p = 0.0012, *Z* = 3.24, *n* = 18 session comparisons from 9 mice, two-tailed sign-rank test). **h** Anatomical location of disPCp from a representative mouse. Each filled dot is a centroid of one disPCp neuron color coded for different temporal clusters. Gray circles are the centroids of other recorded CA1 neurons that were not disPCp.

We next hypothesized that the recruitment of disPCp cells to represent the MA-paired context depended on where those cells fired during the baseline session. Specifically, if a cell fired at a location in baseline that was subsequently visited during drug conditioning, we hypothesized that this cell was more likely to be recruited as disPCp. As the center of the CPP junction was not accessible during conditioning, we used center-firing cells in the test session as a comparison group to test this hypothesis (Fig. 5a, b). In baseline sessions, we observed a subset of place cells that were selectively active in the junction between the two CPP compartments (‘center-firing’ neurons) (Fig. 5b). As visiting the junction location was not possible during the conditioning sessions, in which the drug-context association took place, we hypothesized that ‘center-firing’ neurons in baseline would have a minimal contribution to the encoding of drug-context associations, which could manifest as a lower proportion of ‘center-firing’ neurons classified as disPCp in MA mice. To test this hypothesis, we used a k-means method to group neurons into temporally synchronized clusters using their calcium signals in the baseline session (Fig. 5c, Supplementary Fig. 5, “Methods”). This clustering approach identified a ‘center cluster’ (i.e., center-firing neurons), as well as four clusters with peak ensemble activity at different spatial positions across the CPP compartments (Northeast [NE], Northwest [NW], Southeast [SE], and Southwest [SW]) (Supplementary Fig. 6a–c).

The proportion of neurons assigned to each cluster was roughly equal across animals (Fig. 5d). While disPCp were equally distributed across the five clusters in Ctrl mice, they were not equally distributed in MA mice (Fig. 5e, f). In MA mice, this unequal distribution was visualized by plotting the summed disPCp rate maps according to their cluster assignment (Fig. 5e), which revealed a smaller proportion of disPCp in the center cluster (mean ± SEM, 0.12 ± 0.01) compared to all other clusters (NW: 0.19 ± 0.01, SW: 0.19 ± 0.03, NE: 0.18 ± 0.02, SE: 0.22 ± 0.02) (Fig. 5f). This effect was not due to insufficient neural activity during the conditioning sessions in MA mice (Supplementary Fig. 6d), as during conditioning, center cluster neurons often remapped such that they were maximally active in another spatial location (Fig. 5b). We also did not observe any differences in the number of traversals across the junction between baseline and test sessions (Supplementary Fig. 6e) nor the running speed (Supplementary Fig. 2d). As further validation of this result, we quantified the spatial correlation of place cells in each cluster between spatial maps in baseline and test sessions, as a higher proportion of disPCp decreases this correlation value. As expected, in MA mice, center-clustered neurons showed a higher spatial map correlation compared to all other clusters, consistent with a lower proportion for disPCp in the center cluster compared to all other clusters (Fig. 5g). Interestingly, in MA mice, the anatomical distribution across CA1 of each disPCp cluster was intermingled (Fig. 5h), as demonstrated by the quantification of the pair-wise intra- vs. inter-cluster distances within disPCp (Supplementary Fig. 6f). Together, these results suggest that disPCp depend, in part, on the spatial locations experienced during drug conditioning.

### Drug-context associations primarily affect place cells that are exclusively tuned to position

**Fig. 6:**
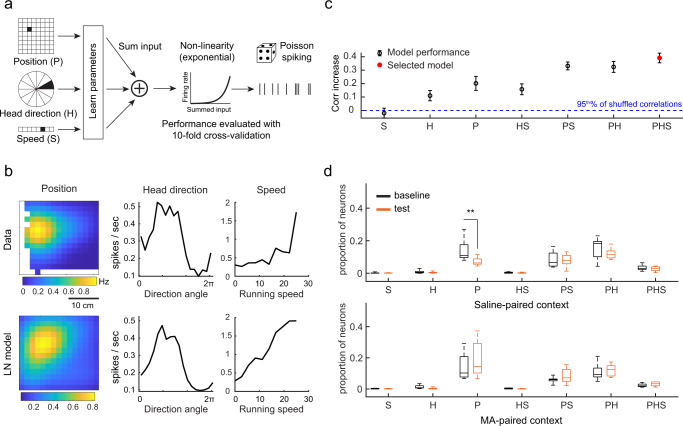
Drug-context associations primarily affect place cells that are exclusively tuned to allocentric position. **a** Schematic of linear-non-linear Poisson (LN) model framework. P represents position, H represents head direction, S represents speed. **b** Calcium event rate tuning curves (top) and model-derived response profiles from an example PHS cell. **c** Example of model performance across all models for the example neuron in (**b**) (mean ± SEM, correlation increase relative to the 95th % of the shuffled correlation, *n* = 10 cross validations). The selected model is indicated in red. **d** Proportion of total neurons for each cell type in baseline and test sessions of MA mice. There is a significant decrease of P-encoding neurons in test compared to baseline, specifically in the saline-paired context (*p* = 0.0039, two-tailed sign-rank test, *n* = 9 mice). For each box plot, the center indicates median, the box indicates 25th and 75th percentiles. The whiskers extend to the most extreme data points without outliers.

Previous studies have shown that neurons in the hippocampus and medial entorhinal cortex can conjunctively respond to an animal’s spatial position, head direction, and speed (i.e., exhibit mixed selectivity)^57–59^ and that task learning impacts mixed-selective hippocampal place cells more than position coding only place cells^58^. To examine whether drug-context associations influenced mixed-selective versus position coding only place cells, we used a linear-non-linear Poisson (LN) model^57^ (Fig. 6a). This approach has the additional benefit of accounting for potential correlations between spatial variables and behavioral variables such as running speed or head direction^57,58^. Using the LN model, we then classified place cells as those that exclusively encoded position versus those that encoded position (P) with speed (S) and/or head direction (HD) (i.e., showed mixed selectivity, Fig. 6b, c)^58^. Consistent with previous studies^58–61^, we identified position coding only (P) and mixed-selective CA1 place cells (PS, PH, PHS), with very few CA1 cells tuned only to speed or head direction (H, S, HS) (Fig. 6b–d and Supplementary Fig. 7). Consistent with our previous analyses, we observed a decreased number of place cells specifically in the saline-paired context in MA mice (Fig. 6d). Interestingly, drug-context associative learning primarily decreased the number of position coding only place cells (P) in the saline-paired context, while the number of mixed-selective place cells (PS, PH, PHS) did not change after drug conditioning (Fig. 6d). These effects were not observed in either Ctrl or sucrose mice (Supplementary Fig. 7). However, in sucrose mice, we observed an increase in the number of position coding only place cells specifically in the sucrose-paired context, which is consistent with previous works that observe place cell over-representation of food reward^44,46,62,63^. This increase in the number of position coding-only place cells in the sucrose-paired context differed from our prior result that considered both position coding only and mixed selective place cells together (Fig. 3e). Nevertheless, in both sets of analyses (Fig. 2e and Supplementary Fig. 7b), MA and sucrose conditioning showed distinct impacts on place cells, with only MA conditioning decreasing the number of place cells in the saline-paired context. Thus together, the LN model-based approach confirmed the different effects that drug versus sucrose conditioning have on place cell coding. Moreover, the model revealed that both drug-context and natural reward associative learning impacted place cells dedicated to encoding the position of the animal more than mixed selective place cells.

## Discussion

Addiction is often viewed as a process of pathological learning. Here, we investigated whether addictive substances could leverage neural circuits of natural learning and memory to achieve maladaptive learning, which may play a role in later drug-seeking behavior. We report that a subset of CA1 place cells (disPCp) correspond to associative learning between drug rewards and environmental context. The emergence of disPCp was consistent across addictive drugs (i.e., methamphetamine and morphine) and not observed in response to natural reward learning nor drug withdrawal. This functionally defined cell class represented the drug-paired context by switching off their activity in the saline-paired context and sharpening their place coding in the drug-paired context. These changes in disPCp activity resulted in a more orthogonalized representation for the two CPP contexts, with the degree to which disPCp remapped between the two contexts correlated with CPP behavior. Further, drug conditioning primarily impacted place cells tuned only to position, and not mixed selective place cells. Together, this work reveals a sub-population of hippocampal CA1 place cells that encode drug-associated contextual information, raising the possibility that future work may be able to target the specific subset of neurons that encode memories of drug use^64–66^.

Previous works have shown that place cell activity can be modulated by spatial locations with a high behavioral significance, such as goal locations associated with rewards (e.g., food or water; for review, see ref. 67). Several phenomena have been observed regarding reward-driven changes to hippocampal place cell coding features. First, place cell firing fields often accumulate near locations associated with reward^44,46,62,63^. The over-representation of reward locations by place cells typically develops through experience, with the firing fields of place cells gradually shifting closer to the reward or goal location over behavioral trials or sessions^35,36,68–70^. This over-representation of reward locations also reflects the activity of a small population of hippocampal neurons dedicated to coding reward^62^. Second, studies have reported elevated activity in existing place cells (i.e., out-of-field firing activity) around reward or goal locations, which is not accompanied by an accumulation of place fields^71–73^. Consistent with previous observations of reward-associated changes in place cells, disPCp developed with experience, and ultimately increased the relative representation of the drug-paired context. Moreover, in the sucrose CPP experiment, we observed an over-representation in the sucrose-paired context in position coding only place cells. Of note, however, in our work, the effect of drug conditioning on place cells (a decreased number of place cells specifically in the saline-paired context) clearly differed from the effect of natural reward conditioning on place cells. Thus, our observations reflect place cell changes specific to learned associations between the reward of a drug and an environmental context, raising the possibility that addictive drugs may usurp the normal hippocampal machinery for learning reward or goal associated locations within an environment^8,74,75^.

Our observation of a large number of place cells that exhibited rate remapping across the two CPP contexts in baseline is consistent with previous works that have shown that place cells can exhibit similar place field locations across geometrically identical contexts or segments of linear tracks^76–79^. Under such conditions, place cells often show homotopic place tuning (i.e., have place fields in similar locations between contexts) with rate remapping, which could support firing rate-based discrimination of different contexts. Interestingly, in the current work, the representation of the two CPP contexts by disPCp became more orthogonal after drug conditioning. One possibility is that this orthogonalization could amplify the differences between the two contexts and facilitate pattern separation, which could support memory encoding and recall of the pairing between the drug and corresponding spatial context. It will be of interest for future work to consider to what degree this orthogonalization in the disPCp representation reflects the distinctness or similarity between the two CPP contexts. An alternative interpretation is that disPCp represent a value-based signal that facilitates a comparison between drug- and saline-paired contexts. However, previous works have found little evidence for the influence of value on hippocampal neural coding^72,80–82^. Thus a more likely interpretation is that the disPCp provide a mechanism for encoding drug-associated contextual information, which then goes on to inform value coding in other brain regions and drive drug-seeking behavior^83,84^.

Of note, while we observed different forms of remapping with drug and saline-paired CPP, our measurements of long-term stability of place cells remained relatively high (median stability values from all conditions >0.5). This differs from previous studies using calcium imaging in freely moving animals, which have reported more dynamic and unstable place cell representations across days (stability values ~ 0.2)^49,85^. One possibility is that the increased long-term place cell stability we observed reflects the geometry or sensory complexity of the CPP environment. Previous works have primarily investigated long-term place cell stability using 1D linear tracks or 1D sensory treadmills, while our mice were running freely in a two chamber 2D environment, which could provide more sensory or landmark input to support stable place maps over longer time periods.

Our observations of drug-driven changes to hippocampal place cell representations almost certainly interface with other brain regions to encode drug-associated contexts and drive drug-seeking behavior. Given the biochemical nature of MA and MO, direct monoamine inputs from locus coeruleus^44,86,87^, VTA^88–90^, and raphe nuclei^91,92^ could all play a role in the emergence of disPCp. Hippocampal representations of drug-associated contexts then likely interface with regions like the nucleus accumbens (NAc), one of the major efferent targets of the hippocampal formation, with hippocampal-NAc circuit interactions playing a key role in spatial context conditioning^9,21,93^. Notably, recent work revealed that cocaine place conditioning increases the functional drive from hippocampal CA1 neurons that encode cocaine-paired locations to NAc medium spiny neurons^17^. Thus, one possibility is that the disPCp route information directly to the NAc to encode drug-associated spatial contexts, with this circuit then potentially provoking future drug-seeking behavior^94^. Another possible long-range target of disPCp are neurons in the lateral septum, a region that plays a critical role in context-induced reinstatement of drug-seeking behaviors^16,23^. Within the hippocampal formation, disPCp may route information to the subiculum^95^, where electrical stimulation has been shown to increase dopamine levels in NAc and trigger cocaine relapse that resembles context-induced drug reinstatement^20,90^. Another potential local efferent target is the medial entorhinal cortex, which innervates the NAc^96,97^ and contains neurons that both encode the position and orientation of an animal that are modulated by reward locations^98,99^. Given the intermingled anatomical distribution of disPCp and collateralization of CA1 neurons, it is likely that drug-associated information encoded by disPCp is broadcast to multiple downstream targets. Nevertheless, further investigation will be needed to identify the full circuit mechanisms by which disPCp interact with known reward circuitry to drive drug-seeking behavior.

## Methods

### Subjects

All procedures were conducted according to the National Institutes of Health guidelines for animal care and use and approved by the Institutional Animal Care and Use Committee at Stanford University School of Medicine. For imaging experiments, Ai94;Camk2a-tTA;Camk2a-Cre (JAX id: 024115 and 005359) mice were used (*n* = 54 total mice in the study). We did not observe obvious epileptiform events in calcium activity or gross abnormal behavior in these mice^100^. Male and female (Ctrl: 3 male and 4 female; MA: 6 male and 4 female; Sucrose: 4 male and 4 female; Sucrose (ctrl): 1 male and 2 female; MO: 8 male and 4 female; CPA MO + saline: 3 male and 3 female; CPA MO + naloxone: 5 male and 3 female) mice were group housed with same-sex littermates until the time of surgery. At the time of surgery, mice were 8–12 weeks old (19–28 g). After surgery mice were singly housed. Mice were kept on a 12-h light/dark cycle and had ad libitum access to food and water in their home cages at all times. All experiments were carried out during the light phase. Due to a limited number of miniscopes and the availability of transgenic mice, up to six mice were used as a cohort for each batch of experiments.

### GRIN lens implantation and baseplate placement

Mice were anesthetized with continuous 1–1.5% isoflurane and head fixed in a rodent stereotax. A three-axis digitally controlled micromanipulator guided by a digital atlas was used to determine bregma and lambda coordinates. To implant the gradient refractive index (GRIN) lens above the CA1 regions of the hippocampus, a 1.8 mm-diameter circular craniotomy was made over the posterior cortex (centered at −2.30 mm anterior/posterior and +1.75 mm medial/lateral, relative to bregma). The dura was then gently removed and the cortex directly below the craniotomy aspirated using a 27- or 30-gauge blunt syringe needle attached to a vacuum pump under constant irrigation with sterile saline. The aspiration removed the corpus callosum above the hippocampal imaging window but left the alveus intact. Excessive bleeding was controlled using a hemostatic sponge that had been torn into small pieces and soaked in sterile saline. As determined using the Allen Brain Atlas (www.brain-map.org/), the unilateral cortical aspiration impacted part of the anteromedial visual area but the procedure left the primary visual area intact. The GRIN lens (0.25 pitch, 0.55 NA, 1.8 mm diameter and 4.31 mm in length, Edmund Optics) was then slowly lowered with a stereotaxic arm to CA1 to a depth of −1.53 mm relative to the measurement of the skull surface at bregma. A skull screw was placed on the contralateral side of the skull surface. Both the GRIN lens and skull screw were then fixed with cyanoacrylate and dental cement. Kwik-Sil (World Precision Instruments) was used to cover the lens at the end of surgery. Two weeks after the implantation of the GRIN lens, a small aluminum baseplate was cemented to the animal’s head on top of the existing dental cement. Specifically, Kwik-Sil was removed to expose the GRIN lens. A miniscope was then fitted into the baseplate and locked in position so that GCaMP6s expressing neurons and visible landmarks, such as blood vessels were in focus in the field of view. After the installation of the baseplate, the imaging window was fixed for the long-term in respect to the miniscope used during installation. Thus, for all imaging experiments, each mouse had a dedicated miniscope. When not imaging, a plastic cap was placed in the baseplate to protect the GRIN lens from dust and dirt.

### Methamphetamine and morphine conditioned place preference (MA or MO CPP)

After mice had fully recovered from the baseplate surgery, they were handled and allowed to habituate to wearing the head-mounted miniscope by freely exploring an open arena for 20 min every day for 1 week. If the animal still showed muscle weakness (as indicated by the difficulty in holding their heads up towards the end of each session) at the end of the first week, they underwent an extra week of habituation. In parallel, animals were also habituated to mock intraperitoneal injections (needle poking) once a day for 4 days.

Conditioned place preference (CPP) sessions took place in a different room from the habituation sessions described in the previous paragraph. This dedicated CPP room contained salient distal visual cues, which were kept constant over the course of the entire experiment. The CPP apparatus consisted of two 25 × 25 cm compartments with distinct colors and visual cues (Fig. 1a). The two compartments could be connected by a sliding door in the middle. The door opening was 6.5 cm wide, so that the mouse could easily run between the two compartments during miniscope recordings. The floors of the CPP compartments were covered with ~500 ml of bedding to facilitate exploration. Mice were first habituated for two days (20 min/day) to the CPP apparatus with the door between the CPP compartments open and the miniscope mounted. Each mouse had its own dedicated miniscope for the entire duration of the CPP experiment, which ensured stable longitudinal recordings and facilitated image alignment across different sessions. Before any experiments started, the image quality for each animal was verified by adjusting the power of the excitation light and the focal plane. These scope parameters then remained fixed on the dedicated miniscope over the course of the entire CPP experiment.

After the 2-day habituation, the pre-baseline session occurred on day 1, in which mice could run freely between the two connected CPP compartments for 20 min (with imaging). On day 2, the baseline session, the same experiment was repeated and the behavioral data used to assess the animals’ naturally preferred context (i.e., the compartment where the animal spent more time). Prior to the experiments, we defined an exclusion threshold, in which we would exclude any mouse that spent more than 75% of their total time in one compartment in the baseline session; however, no mice reached this threshold. The subsequent conditioning sessions (3 sets, 6 days of pairings) were performed by confining the animal in one of the CPP compartments for 45 min immediately after a saline or drug administration. During the 45 min, we only imaged from the 15th–30th minute in each mouse. For each set of conditioning sessions, saline was paired in the preferred context on the first conditioning day and the drug paired (MA at 2 mg/kg or MO at 20 mg/kg, injected intraperitoneally) in the non-preferred context on the second conditioning day. This conditioning design was trying to align drug pairings with animals’ internal state and avoid potential ceiling effects for the exploration time^17,19,51,101–105^. In addition, this design maximumly balanced animals’ spatial coverage between the two CPP contexts, which is critical for place cell analyses (Supplementary Fig. 2a). This conditioning process was repeated 3 times in total such that each animal received 3 saline pairings and 3 drug pairings. Similarly, in control (Ctrl) mice, saline was paired in both CPP contexts, starting with the preferred context. Note that for MA and MO mice, the naturally preferred context was equivalent to the saline-paired context and the non-preferred context was equivalent to the drug-paired context. Twenty-four hours after the last conditioning session, animals were put back into the CPP environment with the two compartments connected again for 20 min with imaging to assess their post-conditioning preferences (defined as test 1). To assess whether any drug-induced preference was long-lasting, we performed a 2nd post-conditioning test (defined as test 2) 5 days after test 1. The behavioral CPP score was defined as the time that the animal spent in the drug-paired context of the CPP apparatus in a test session minus the time it spent in the same context in the baseline session.

### Sucrose conditioned place preference (Sucrose CPP)

Sucrose CPP was performed as previously described^106^, with modifications. In brief, mice were mildly food restricted to ~90–95% of their body weight. Baseline sessions were performed similar to baseline sessions in MA CPP. To minimize the total length of food deprivation, for animal welfare reasons, we accelerated our CPP protocol for the conditioning by using two pairings per day (AM and PM) for 4 days (we only imaged on the first three days). On each day, mice explored their preferred context with drops of water for 20 min (20 μl per min at random locations). Next, mice explored the non-preferred context with drops of 20% sucrose solution for 20 min (20 μl per min at random locations). Sucrose Ctrl mice received water in both contexts during conditioning. One and three days after the conditioning, two test sessions were performed to assess an animal’s preference change.

### Morphine conditioned place aversion (CPA)

Morphine CPA was performed as previously described^107^. Baseline sessions were performed similar to baseline sessions in MA CPP. To establish morphine dependence, mice received a daily intraperitoneal injection of morphine in their home cage for 5 consecutive days. Doses escalated from 10, 20, 30, 40, and 50 mg/kg. On the conditioning day, mice received another 50 mg/kg of morphine first. Two hours after this injection, mice received a single dose of naloxone (5 mg/kg, i.p.) and were put immediately into their naturally preferred context for 20 min (MO + naloxone). For the control condition, mice received a saline injection instead of naloxone (MO + saline). Somatic withdraw signs (jump and rearing) were manually quantified offline. One and three days after the conditioning, two test sessions were performed to assess an animal’s preference change.

### Histology

After the imaging experiment was concluded, mice were deeply anesthetized with isoflurane and transcardially perfused with 5 ml of phosphate-buffered saline (PBS), followed by 25 ml of 4% paraformaldehyde-containing phosphate buffer. The brain was removed and left in 4% paraformaldehyde overnight. The next day, samples were transferred to 30% sucrose in PBS. At least 24 h later, the brain was sectioned coronally into 40-µm-thick samples using a cryostat. Sections were mounted and cover-slipped with antifade mounting media with DAPI (Vectashield). Brain slice images were acquired using a ZEISS Axio Imager 2 fluorescence microscope under ×10 or ×20 magnification for both DAPI and GFP channels.

### Miniscope imaging data acquisition and initial batch processing

Technical details for the custom-constructed miniscopes and general processing analyses are described in refs. 48, 50 and at miniscope.org. Briefly, this head-mounted scope had a mass of about 3 g and a single, flexible coaxial cable to carried power, control signals, and imaging data to custom open source Data Acquisition (DAQ) hardware and software. In our experiments, we used Miniscope V3, which had a 700 μm × 450 μm field of view with a resolution of 752 pixels × 480 pixels (~1 μm per pixel). Acquired data was packaged by the electronics to comply with the USB video class (UVC) protocol. The data was then transmitted via a Super Speed USB to a PC running custom DAQ software. The DAQ software was written in C++ and used Open Computer Vision (OpenCV) libraries for image acquisition. Images were acquired at ~30 frames per second (fps) and recorded to uncompressed.avi files. The DAQ software also recorded the simultaneous behavior of the mouse through a high-definition webcam (Logitech) at ~30 fps, with time stamps applied to both video streams for offline alignment.

Miniscope videos of individual sessions were first concatenated and down-sampled by a factor of 2 using custom MATLAB scripts, then motion corrected using the NoRMCorre MATLAB package^108^. To align miniscope videos across different sessions for the entire CPP experiment, we applied an automatic 2D image registration method (github.com/fordanic/image-registration) with rigid x–y translations according to the maximum intensity projection images for each session. The registered videos for each animal were then concatenated together in chronological order to generate a combined data set for extracting calcium activity. To extract the calcium activity from the large combined data set (>10 GB), we used the Sherlock HPC cluster hosted by Stanford University to process the data across 8–12 cores and 600–700 GB of RAM. While processing this combined data set required significant computing resources, it enhanced our ability to track cells across sessions from different days (Fig. 1). This process made it unnecessary to perform individual footprint alignment or cell registration across sessions.

To extract an individual neuron’s calcium activity, we adopted a method of extended constrained non-negative matrix factorization for endoscopic data (CNMF-E)^54^. CNMF-E is based on the CNMF framework^109^, which enables simultaneous denoising, deconvolving and demixing of calcium imaging data. A key feature includes modeling the large, rapidly fluctuating background, allowing good separation of single-neuron signals from background, and separation of partially overlapping neurons by taking a neuron’s spatial and temporal information into account (see ref. 54 for details). After iteratively solving a constrained matrix factorization problem, CNMF-E extracted the spatial footprints of neurons and their associated temporal calcium activity. Specifically, the first step of estimating a given neuron’s temporal activity (a scaled version of dF/F, an oft-used metric in calcium imaging studies) was to compute the weighted average of fluorescence intensities after subtracting the temporal activity of other neurons in the given neuron’s region of interest. A deconvolution algorithm called OASIS^56^ was then applied to obtain the denoised neural activity and deconvolved spiking activity, as illustrated in Supplementary Fig. 1. These extracted calcium signals for the combined data set were then split back into each session according to their individual frame numbers.

The position and speed of the animal were determined by applying a custom MATLAB script to the animal’s behavioral tracking video. Time points at which the speed of the animal was lower than 2 cm/s were identified and excluded them from further analysis. We then used linear interpolation to temporally align the position data to the calcium imaging data.

To further validate the quality of cross-session alignment using the image registration, we analyzed the maximum projected images from each session after the registration with the colocalization analysis available via NIH ImageJ and the JACoP plugin^110^ (Supplementary Fig. 1). In brief, the two images were background subtracted and a threshold automatically applied based on the Coste’s approach. A cytofluorogram was then plotted for each pixel pair based on their intensity. A Pearson’s coefficient can then be derived by calculating the best-fitting regression line on the cytofluorogram. To test the statistical significance of the colocalization analysis, we used the Coste’s approach to randomly shuffle pixel blocks for one of the images 1000 times and obtained a distribution of concomitantly calculated shuffled coefficients. The 95th percentile of the shuffled distribution were then determined as the significant threshold (Supplementary Fig. 1).

### Position-matching for comparisons of cell activity across sessions and CPP compartments

Analyses that compared hippocampal neuronal activity across different sessions (longitudinal comparisons) or across the two CPP compartments within the same session (transverse comparisons) could be influenced by biases in the animal’s spatial occupancy, particularly due to the CPP-related shift in spatial preference. First, we assessed the spatial coverage of animals in baseline and test sessions. Spatial coverage was quantified as the percentage of spatial bins (bin size = 1.8 × 1.8 cm) that an animal physically visited within each session. We found most animals had stable spatial coverage (>90%) across sessions and between the two CPP contexts, despite the place preference change (Supplementary Fig. 3a). To further circumvent the effect of differences in occupancy on our analyses, we implemented a position-matched down-sampling protocol^99^ when performing longitudinal or transverse comparisons of place cell activity. For down-sampling, we first binned the spatial arena into 1.8 × 1.8 cm non-overlapping bins. We then computed the number of position samples (frames) observed in each spatial bin for the to-be-matched sessions. Finally, the number of samples in each corresponding spatial bin was down-sampled by randomly removing position samples, and the corresponding neural activity, from the session with greater occupancy (Supplementary Fig. 2b, c). Due to the stochastic nature of the down-sampling process, we repeated this procedure 50 times (unless otherwise specified) for each cell, and the final value for each cell was calculated as the average of all 50 iterations. This final value was then used to obtain the reported means or perform statistic comparisons. This protocol was applied to our analyses for all the within-subject comparisons (both longitudinal and transverse). Specific details for each analysis are described in the corresponding methods and figure legends.

### Place cell analyses

#### Calculation of spatial rate maps

After we obtained the deconvolved spiking activity of neurons, we extracted and binarized the effective neuronal calcium events from the deconvolved spiking activity by applying a threshold (3 × standard deviation of all the deconvolved spiking activity for each neuron). The position data was sorted into 1.8 × 1.8 cm non-overlapping spatial bins. The spatial rate map for each neuron was constructed by dividing the total number of calcium events by the animal’s total occupancy in a given spatial bin. The rate maps were smoothed using a 2D convolution with a Gaussian filter that had a standard deviation of 2.

#### Spatial information and identification of place cells

To quantify the information content of a given neuron’s activity, we calculated spatial information scores in bits/spike (each calcium event is treated as a spike here) for each neuron according to the following formula^111^,

1 $${{{{{\rm{Bits}}}}}}/{{{{{\rm{spike}}}}}}=\mathop{\sum }\limits_{{{{{{\rm{i}}}}}}=1}^{{{{{{\rm{n}}}}}}}{{{{{{\rm{P}}}}}}}_{{{{{{\rm{i}}}}}}}\frac{{{{\lambda }}}_{{{{{{\rm{i}}}}}}}}{{{\lambda }}}{{{\log }}}_{2}\frac{{{{\lambda }}}_{{{{{{\rm{i}}}}}}}}{{{\lambda }}},$$

where ${{{{{{\rm{P}}}}}}}_{{{{{{\rm{i}}}}}}}$ is the probability of the mouse occupying the i-th bin for the neuron, ${{{\lambda }}}_{{{{{{\rm{i}}}}}}}$ is the neuron’s unsmoothed event rate in the i-th bin, while ${{\lambda }}$ is the mean rate of the neuron across the entire session. Bins with total occupancy time of <0.1 s were excluded from the calculation. To identify place cells, the timing of calcium events for each neuron was circularly shuffled 1000 times and spatial information (bits/spike) recalculated for each shuffle. This generated a distribution of shuffled information scores for each individual neuron. The value at the 95th % of each shuffled distribution was used as the threshold for classifying a given neuron as a place cell, and we excluded cells with an overall mean calcium event rate <0.1 Hz. This threshold was roughly equal to the 5th % of the mean event rate distribution for all neurons.

To avoid the potential influence of drug-induced change in occupancy on identifying place cells, we classified place cells by applying the position-matching protocol (described in the previous section) in addition to the above shuffling procedure. Namely, the full shuffling procedure was repeated for 20 times, each time on down-sampled neural activity obtained to match the position occupancy of the animal across the two CPP compartments. From these 20 repetitions, we calculated a place cell agreement index (PCI) for each neuron as

2 $${{{{{\rm{PCI}}}}}}=\mathop{\sum }\limits_{{{{{{\rm{i}}}}}}=1}^{20}{{{{{\rm{indicator}}}}}}({{{{{{\rm{cell}}}}}}}_{{{{{{\rm{iter}}}}}}={{{{{\rm{i}}}}}}}={{{{{\rm{place\; cell}}}}}})/20,$$

where indicator() is a function that returns 1 when the equation inside the parentheses holds, and 0 otherwise; i is the number of iterations (iter). We chose PCI ≥ 0.3 as the threshold for classifying a neuron as a place cell, based on both the visual inspection of place fields and the resulting proportion of place cells (~70% of total neurons, union of the place cells from both CPP compartments), which was similar to conventional tetrode recordings from CA1^112,113^. Note that compared with the regular shuffling method that does not perform position matching, the current approach results in slightly fewer cells classified as place cells. Although the place cells classified and used in the current study were all obtained through this position matching method, the general findings regarding the number of place cells described in the results (e.g., Fig. 2) are robust and remain the same even without position matching.

#### Rate map spatial correlation, cross-session stability, mean, and peak Ca^2+^ event rate

We applied the position matching protocol in our measures of rate map spatial correlation, cross-session stability, mean and peak Ca^2+^ event rate of place cells. For each matching iteration, we first computed occupancy-matched rate maps across different sessions or contexts (Supplementary Fig. 2b, c). Spatial correlation or cross-session stability was calculated as the Pearson’s correlation coefficient between the occupancy-matched rate maps. Mean Ca^2+^ event rate was measured as the number of spikes in the occupancy-matched rate maps divided by the summed matched-occupancy time. Peak Ca^2+^ event rate was measured as the maximum of the occupancy-matched rate map. Final values for these metrics were obtained by averaging all the 50 matching iterations.

#### Quantification of place field size

We only measured place fields in neurons classified as place cells in a given environment. To measure the size of a given place field, the occupancy-matched rate map was first binarized by applying a threshold of 50% of the peak event rate for the rate map. The place fields were then identified and extracted as connected objects from the binarized rate map. For place cells with more than one place field, we used the largest place field as the measurement. Similarly, final values were obtained by averaging all the 50 matching iterations.

### Reconstructing the mouse’s position using a naive Bayes classifier

We used a naive Bayes classifier to estimate the probability of animal’s location given the activity of all the recorded neurons. Speed filtered (>2 cm/s), thresholded (3 × standard deviation of all the deconvolved spiking activity for each neuron), and binarized deconvolved spike activity (neuron.S in CNMF-E) from all neurons were first binned into non-overlapping time bins of 0.8 s. This time bin width was selected based on the overall decoding performance among all the bin width tested ranging from 0.2 to 1.6 s. The M × N spike data matrix, where M is the number of time bins and N is the number of neurons, was then used to train the decoder with an M × 1 vectorized location labels. The posterior probability of observing the animal’s position Y given neural activity X can then be inferred from the Bayes rule as:

3 $${{{{{\rm{P}}}}}}\big({{{{{\rm{Y}}}}}}={{{{{\rm{y}}}}}}|{{{{{{\rm{X}}}}}}}_{1},\,{{{{{{\rm{X}}}}}}}_{2}\ldots,\,{{{{{{\rm{X}}}}}}}_{{{{{{\rm{N}}}}}}}\big)=\frac{{{{{{\rm{P}}}}}}\big({{{{{{\rm{X}}}}}}}_{1},\,{{{{{{\rm{X}}}}}}}_{2},\ldots,\,{{{{{{\rm{X}}}}}}}_{{{{{{\rm{N}}}}}}}|{{{{{\rm{Y}}}}}}={{{{{\rm{y}}}}}}\big){{{{{\rm{P}}}}}}({{{{{\rm{Y}}}}}}={{{{{\rm{y}}}}}})}{{{{{{\rm{P}}}}}}\left({{{{{{\rm{X}}}}}}}_{1},\,{{{{{{\rm{X}}}}}}}_{2},\,\ldots,\,{{{{{{\rm{X}}}}}}}_{{{{{{\rm{N}}}}}}}\right)},$$

where X = (X_1_, X_2_, … X_N_) is the activity of all neurons, y is one of the spatial bins that the animal visited at a given time, and P(Y = y) is the prior probability of the animal being in spatial bin y. We used an empirical prior as it showed slightly better performance than a flat prior. P(X_1_, X_2_, …, X_N_) is the overall firing probability for all neurons, which can be considered as a constant and does not need to be estimated directly. Thus, the relationship can be simplified to

4 $${{{{{\rm{P}}}}}}\big({{{{{\rm{Y}}}}}}={{{{{\rm{y}}}}}}|{{{{{{\rm{X}}}}}}}_{1},{{{{{{\rm{X}}}}}}}_{2}\ldots,{{{{{{\rm{X}}}}}}}_{{{{{{\rm{N}}}}}}}\big)\propto {{{{{\rm{P}}}}}}({{{{{\rm{Y}}}}}}={{{{{\rm{y}}}}}})\mathop{\prod }\limits_{{{{{{\rm{i}}}}}}=1}^{{{{{{\rm{N}}}}}}}{{{{{\rm{P}}}}}}\big({{{{{{\rm{X}}}}}}}_{{{{{{\rm{i}}}}}}}|{{{{{\rm{Y}}}}}}={{{{{\rm{y}}}}}}\big),$$

5 $$\hat{{{{{{\rm{y}}}}}}}={{\arg }}\mathop{{{\max }}}\limits_{{{{{{\rm{y}}}}}}}{{{{{\rm{P}}}}}}({{{{{\rm{Y}}}}}}={{{{{\rm{y}}}}}})\mathop{\prod }\limits_{{{{{{\rm{i}}}}}}=1}^{{{{{{\rm{N}}}}}}}{{{{{\rm{P}}}}}}\big({{{{{{\rm{X}}}}}}}_{{{{{{\rm{i}}}}}}}|{{{{{\rm{Y}}}}}}={{{{{\rm{y}}}}}}\big),$$

where $\hat{{{{{{\rm{y}}}}}}}$ is the animal’s predicted location, based on which spatial bin has the maximum probability across all the spatial bins for a given time. To estimate P(X_i_ | Y = y), we applied the built-in function of MATLAB fitcnb() to fit a multinomial distribution using the bag-of-tokens model with Laplace smoothing, which gave an estimation of distribution parameter for each neuron X_i_ at the given spatial bin y as:

6 $${{{{{\rm{\theta }}}}}}\left({{{{{{\rm{X}}}}}}}_{{{{{{\rm{i}}}}}}}|{{{{{\rm{Y}}}}}}={{{{{\rm{y}}}}}}\right)=\frac{\mathop{\sum }\nolimits_{{{{{{\rm{j}}}}}}=1}^{{{{{{\rm{M}}}}}}}\big({{{{{{\rm{X}}}}}}}_{{{{{{\rm{i}}}}}}}=\,{{{{{{\rm{X}}}}}}}_{{{{{{\rm{i}}}}}}}|{{{{{{\rm{Y}}}}}}}^{({{{{{\rm{j}}}}}})}={{{{{\rm{y}}}}}}\big)+1}{\mathop{\sum }\nolimits_{{{{{{\rm{k}}}}}}=1}^{{{{{{\rm{N}}}}}}}\mathop{\sum }_{{{{{{\rm{j}}}}}}=1}^{{{{{{\rm{M}}}}}}}\big({{{{{{\rm{X}}}}}}}_{{{{{{\rm{i}}}}}}}={{{{{{\rm{X}}}}}}}_{{{{{{\rm{k}}}}}}}|{{{{{{\rm{Y}}}}}}}^{({{{{{\rm{j}}}}}})}={{{{{\rm{y}}}}}}\big)+{{{{{\rm{N}}}}}}},$$

where, regardless of the Laplace smoothing, the numerator is the total number of spikes of neuron X_i_ for the time bins that the animal is at location y, and the denominator is the total number of spikes of all the neurons for the time bins that the animals is at location y. In addition, the above equation was weighted such that the normalized weights within a location bin sum to the prior probability for that location bin. This multinomial-based model offers high decoding accuracy, which is important as we are investigating the function of a small population of neurons.

In addition, to reduce occasional erratic jumps in position estimates, we implemented a 2-step Bayesian method by introducing a continuity constraint^114^, which incorporated information regarding the decoded position in the previous time step and the animal’s running speed to calculate the probability of the current location y. The continuity constraint for all the spatial bins Y at time t followed a 2D gaussian distribution centered at position y_t-1_, which can be written as:

7 $${{{{{\mathcal{N}}}}}}\left({{{{{{\rm{y}}}}}}}_{{{{{{\rm{t}}}}}}-1},\,{{{{{{\rm{\sigma }}}}}}}_{{{{{{\rm{t}}}}}}}^{2}\right)=c*{{\exp }}\left(\frac{{-{{{{{\rm{||}}}}}}{{{{{{\rm{y}}}}}}}_{{{{{{\rm{t}}}}}}-1}-{{{{{\rm{Y||}}}}}}}^{2}}{{2{{{{{\rm{\sigma }}}}}}}_{{{{{{\rm{t}}}}}}}^{2}}\right),$$

8 $${{{{{{\rm{\sigma }}}}}}}_{{{{{{\rm{t}}}}}}}=a{{{{{{\rm{v}}}}}}}_{{{{{{\rm{t}}}}}}},$$

where *c* is a scaling factor and v_t_ is the instantaneous speed of the animal between time t−1 and t. v_t_ is scaled by $a$, which is empirically selected as 2.5. The final reconstructed position with 2-step Bayesian method can be further written as:

9 $${\hat{{{{{{\rm{y}}}}}}}}_{2{{{{{\rm{step}}}}}}}={{\arg }}\mathop{\max }\limits_{{{{{{\rm{y}}}}}}}{{{{{\mathscr{N}}}}}}({{{{{{\rm{y}}}}}}}_{{{{{{\rm{t}}}}}}-1},\,{{{{{{\rm{\sigma }}}}}}}_{{{{{{\rm{t}}}}}}}^{2}){{{{{\rm{P}}}}}}({{{{{\rm{Y}}}}}}={{{{{\rm{y}}}}}})\mathop{\prod }\limits_{{{{{{\rm{i}}}}}}=1}^{{{{{{\rm{N}}}}}}}{{{{{\rm{P}}}}}}\big({{{{{{\rm{X}}}}}}}_{{{{{{\rm{i}}}}}}}|{{{{{\rm{Y}}}}}}={{{{{\rm{y}}}}}}\big).$$

Decoded vectorized positions were then mapped back onto 2D space. The decoding error was calculated as the mean Euclidean distance between the decoded position and the animal’s true position, across all time bins. The overall improvement gained by implementing the 2-step Bayesian method was only ~5–10%, which indicated that the method did not introduce any direct information regarding the animal’s current location to the decoder.

To compare position decoding performance across mice, we randomly down-sampled the number of neurons such that they matched across all mice (*n* = 150). We performed this down-sampling 50 times and trained the decoder and generated position predictions for each iteration. The final result for a given mouse was then calculated as the average decoding performance across the 50 iterations. To examine the decoder’s performance using shuffled data, we circularly shuffled the neural data 100 times, shifting the spike times by 5–95% of the total data length randomly (i.e., 60–1140 s for a 20-min recording). The final result was then calculated as the average decoding performance across the 100 shuffles. To compare position decoding performance between the two CPP compartments, data for both training and predicting were matched for occupancy 50 times. The final result for a given compartment was then calculated as the average decoding performance across the 50 iterations.

For the knock-out (KO) decoding analyses, we replaced the neural activity of cells that were ‘knocked out’ with vectors of zeroes. This knock-out procedure was only applied to the data we used for predicting position locations, not for training, as ablating neurons directly from the training data will result in the model learning to compensate for the missing information^115^. In addition, for the KO decoding analyses, the training data set was subject to the position matching protocol. The final result for each mouse was then calculated as the averaged decoding performance across all of the position matching iterations. For the random KO condition, we randomly selected the same number of neurons as in the KO condition for each iteration. For conditions in which the training and prediction data were both from the baseline or test sessions, we used the session with a lower time bias between the two compartments as the training data, which allowed us to use the session with the maximum amount of training data after position matching between the two CPP compartments. As in the regular decoding analysis, the KO decoding analysis provided reconstructed/predicted positions for an animal based on the neural activity; the CPP time could then be reconstructed from this position result. For KO analyses in Fig. 4h–j, the decoder was trained using the baseline data, and rtPCp (retained place cells on the preferred side) were randomly selected for KO to match the number of disPCp from the test sessions data before making predictions. Both training and prediction data were occupancy matched between the two compartments. We then calculated the final decoding error for the predicted position by averaging the decoding errors from all the position matching iterations for each compartment.

### Temporal clustering with k-means

#### Identification of temporal clusters

To cluster neurons according to the temporal information of their calcium signals, we employed a consensus k-means clustering method and calculated a consensus matrix that measured how frequently two samples were clustered together in multiple clustering runs with randomly sub-sampled data. We used the denoised calcium trace (neuron.C in CNMF-E, Supplementary Fig. 1e, blue trace) as a measure of neural activity and filtered the signal with a noise level threshold of 2 x (neuron.C_raw – neuron.C) for each neuron. We then computed the consensus matrix using sub-sampled data from the baseline session for 100 iterations. For each iteration, the calcium data from all the neurons was randomly sub-sampled at 90% of the total frame length, and neurons were partitioned into 5 groups (see determining optimal K below) using k-means clustering by calculating the pair-wise Pearson’s correlation coefficient from the sub-sampled calcium data. In each k-means run, there were 10 repeated clustering (replicates) using new initial cluster centroid positions. A consensus matrix was then obtained upon the completion of all the iterations by calculating the frequency with which two neurons were grouped together. Neuron pairs that showed the same cluster assignment across the highest number of iterations had a high consensus index value. On the other hand, neuron pairs that rarely clustered together had a low consensus index value. The final cluster was then determined using a hierarchical clustering method with complete linkage on the consensus matrix.

#### Determining the optimal number of clusters (K)

To estimate the optimal K value, we chose to search from 3 to 9 clusters. As we estimated the optimal K using either the local minima or maxima from the measurements described below, we expanded our K value search to range from 2 to 10 clusters for calculating the optimal K value. To determine the optimal K, we examined the performance of the K-clustered consensus matrix by visualizing the heatmap reorganized by linkage (Supplementary Fig. 5). The consensus matrix can also be visualized by plotting it as a cumulative distribution function (CDF), as shown in Supplementary Fig. 5b. In the case of perfect clustering, the value of the consensus matrix will be either 0 or 1. Thus, the corresponding CDF would follow a Bernoulli distribution and the curve would be flat for intermediate values. Based on this principle, we employed a metric called the Proportion of Ambiguous Clustering (PAC) to estimate the optimal K^116^. PAC is defined as the fraction of sample pairs with a consensus index value between [0.1, 0.9]. A low PAC value indicates the CDF curve is flat in the middle, thus allowing inference of the optimal K by identifying the lowest PAC (Supplementary Fig. 5c). In addition to PAC, we also implemented a cophenetic correlation based measurement to infer the optimal K^117^. This measurement computes the Pearson’s correlation between the distance of neuron pairs in the consensus matrix and the cophenetic distance of neuron pairs obtained from the dendrogram tree used to reorder the consensus matrix by linkage. Thus, the cophenetic correlation measures how faithfully the dendrogram tree represents the distance between neuron pairs. A cophenetic correlation equal to 1 indicates a perfect consensus matrix. We plotted the cophenetic correlation as a function of K, with a range from 2 to 10, and selected the local maxima of cophenetic correlation as the optimal K (Supplementary Fig. 5d). As shown in Supplementary Fig. 5e, f, measurements from both methods gave similar inferences, pointing to the optimal K = 5.

#### Template matching method for sorting temporally defined clusters according to their spatial firing patterns

To compare temporally defined clusters across different animals, we took advantage of the ensemble spatial firing patterns for each temporally defined cluster and sorted them in into the following five groups: SW, southwest; SE, southeast; NW, northwest; NE, northeast, and center. These directions denoted the location of the peak in the ensemble activity in respect to the CPP environment. For each cluster, the peak of the ensemble activity in the two CPP compartments were always at a similar location (Supplementary Fig. 6) except for the center cluster, which only has a single peak at the junction between the two compartments. To perform unbiased and automated sorting, we developed a template matching method and computed the Pearson’s correlation between the ensemble spatial firing maps for each cluster and standard gaussian templates for each direction (Supplementary Fig. 6). Each standard template contains two simulated Gaussian fields at homotopic positions across the two compartments (Supplementary Fig. 6a). The field akin to the CPP midline had a variance of 12.5 cm while the field away from the midline had a variance of 25 cm. To avoid aberrant matching performance, the center group, which can be unambiguously identified, was held out during this matching process. The final matching result gave a 4 × 4 correlation matrix (Supplementary Fig. 6c), which we used to sort the ensemble activity of each cluster by assigning it to the group with the highest correlation. In most animals, temporal clusters fell into one of these non-overlapping groups with an unambiguous ordering. Occasionally, group ordering with maximum summed correlation was used if there was ambiguity in the group assignment.

#### Measuring paired-wise anatomical distances

To measure the paired-wise anatomical distance for all disPCp in each mouse, we calculated the Euclidian distance between the centroid locations of each disPCp pair under the imaging window for each mouse. The centroid location of the neuron was obtained from the CNMF-E framework (neuron.centroid), denoting the center coordinates for each ROI contour. For each disPCp, we quantified an averaged intra- vs. inter-cluster distances based on the cluster assignment for all disPCp. The final result for each cluster was averaged across all disPCp that belonged to the same cluster. We expected that the inter-cluster distance would be larger than the intra-cluster distance, if functionally-defined disPCp clusters are anatomically clustered.

### Linear-non-linear Poisson (LN) model

The LN model is a generalized linear model (GLM) framework which allows unbiased identification of functional cell types encoding multiplexed navigational variables. This framework was described in a previous publication^57^ and here, we applied the same method to our calcium imaging data in the hippocampus. Briefly, seven models were built in the LN framework, including position (P), head direction (H), speed (S), position & head direction (PH), position & speed (PS), head direction & speed (HS), and position & head direction & speed (PHS). For each model, the dependence of spiking on the corresponding variable(s) was quantified by estimating the spike rate (r_t_) of a neuron during time bin t as an exponential function of the sum of variable values (for example, the animal’s position at time bin t, indicated through an ‘animal-state’ vector) projected onto a corresponding set of parameters (Fig. 6a). This can be mathematically expressed as

10 $${{{{{\rm{r}}}}}}=\frac{{{\exp }}(\mathop{\sum}\limits_{{{{{{\rm{i}}}}}}}{{{{{{\rm{X}}}}}}}_{{{{{{\rm{i}}}}}}}^{{{{{{\rm{T}}}}}}}{{{{{{\rm{w}}}}}}}_{{{{{{\rm{i}}}}}}})}{{{{{{\rm{dt}}}}}}}$$

where r is a vector of firing rates for one neuron over T time points, i indexes the variable (i ∈ [P, H, S]), X_i_ is the design matrix in which each column is an animal-state vector xi for variable i at one time bin, w_i_ is a column vector of learned parameters that converts animal-state vectors into a firing rate contribution, and dt is the time-bin width.

We used thresholded deconvolved spikes (neuron.S in CNMFe) as the neuron spiking data with a time-bin width equal to 500 ms. To achieve sufficient training data for modeling, we concatenated the data from two baseline sessions as a single session and the two test sessions as a single session. The design matrix contained the animal’s behavioral state, in which we binned position into 1.8 cm^2^ bins, head direction into 20-degree bins, and speed into 2 cm/s bins. Each vector in the design matrix denotes a binned variable value. All elements of this vector are 0, except for a single element that corresponds to the bin of the current animal-state. To learn the variable parameters w_i_, we used the built-in fminunc function in MATLAB to maximize the Poisson log-likelihood of the observed spike train (n) given the model spike number (r × dt) and under the prior knowledge that the parameters should be smooth. Model performance for each cell is computed as the increase in Pearson’s correlation (between the predicted and the true firing rates) of the model compared to the 95th % of shuffled correlations (true firing rate was circularly shuffled for 500 times). Performance was quantified through tenfold cross-validation, where each fold is a random selection of 10% of the data. To determine the best-fitted model for a given neuron, we used a heuristic forward-search method that determines whether adding variables significantly improves model performance (*p* < 0.05 for a one-sided sign-rank test, *n* = 10 cross-validation folds).

### Statistical analysis

All the analyses and statistical tests were performed using MATLAB (2017b and 2020a). Data are presented as mean ± SEM or median ± interquartile range (IQR), as indicated. Parametric tests were used for datasets with a normal distribution, as determined by the Shapiro–Wilk test, which included an animal’s CPP score, running speed, center time, and the proportions of cells. For statistical comparisons between groups, an unpaired *t*-test was used to compare two groups for normally distributed results; otherwise, a Wilcoxon rank-sum test was used. For statistical comparisons across more than two groups, one-way ANOVA and related multiple comparison tests were used. For paired statistical comparisons, a paired *t*-test was used if the data followed a normal distribution; otherwise, a Wilcoxon sign-rank test was used. All tests were two-tailed unless otherwise specified. All the statistical tests were performed based on each animal or each session, as indicated in the corresponding figure legend. In all experiments, the level of statistical significance was defined as *p* ≤ 0.05.

### Reporting summary

Further information on research design is available in the Nature Research Reporting Summary linked to this article.

## Supplementary information

Supplementary Information

Description of Additional Supplementary Files

Supplementary Movie 1

Supplementary Movie 2

Supplementary Movie 3

Reporting Summary

## Data Availability

The datasets generated in the current study are available on Mendeley Data: 10.17632/p8gh7wk9z3.1 Source data are provided with this paper.

## Source data

Source Data

## Code availability

Custom MATLAB code generated in this study is available at: https://github.com/yanjuns/Sun_Giocomo_2022_NComms.

## References

[1] Hunt WA, Barnett LW, Branch LG (1971). Relapse rates in addiction programs. J. Clin. Psychol..

[2] Wikler A (1973). Dynamics of drug dependence. Implications of a conditioning theory for research and treatment. Arch. Gen. Psychiatry.

[3] O’Brien CP, Childress AR, McLellan AT, Ehrman R (1992). Classical conditioning in drug-dependent humans. Ann. N. Y Acad. Sci..

[4] Crombag HS, Shaham Y (2002). Renewal of drug seeking by contextual cues after prolonged extinction in rats. Behav. Neurosci..

[5] Fuchs RA (2005). The role of the dorsomedial prefrontal cortex, basolateral amygdala, and dorsal hippocampus in contextual reinstatement of cocaine seeking in rats. Neuropsychopharmacology.

[6] Rubio FJ (2015). Context-induced reinstatement of methamphetamine seeking is associated with unique molecular alterations in Fos-expressing dorsolateral striatum neurons. J. Neurosci..

[7] Crombag HS, Bossert JM, Koya E, Shaham Y (2008). Review. Context-induced relapse to drug seeking: a review. Philos. Trans. R. Soc. Lond. B Biol. Sci..

[8] Hyman SE (2005). Addiction: a disease of learning and memory. Am. J. Psychiatry.

[9] Ito R, Robbins TW, Pennartz CM, Everitt BJ (2008). Functional interaction between the hippocampus and nucleus accumbens shell is necessary for the acquisition of appetitive spatial context conditioning. J. Neurosci..

[10] Ito R, Robbins TW, McNaughton BL, Everitt BJ (2006). Selective excitotoxic lesions of the hippocampus and basolateral amygdala have dissociable effects on appetitive cue and place conditioning based on path integration in a novel Y-maze procedure. Eur. J. Neurosci..

[11] Fuchs RA, Eaddy JL, Su Z-I, Bell GH (2007). Interactions of the basolateral amygdala with the dorsal hippocampus and dorsomedial prefrontal cortex regulate drug context-induced reinstatement of cocaine-seeking in rats. Eur. J. Neurosci..

[12] White NM, Chai SC, Hamdani S (2005). Learning the morphine conditioned cue preference: Cue configuration determines effects of lesions. Pharmacol. Biochem. Behav..

[13] White NM (1996). Addictive drugs as reinforcers: multiple partial actions on memory systems. Addiction.

[14] McDonald RJ, White NM (1993). A triple dissociation of memory systems: hippocampus, amygdala, and dorsal striatum. Behav. Neurosci..

[15] Everitt BJ, Robbins TW (2005). Neural systems of reinforcement for drug addiction: from actions to habits to compulsion. Nat. Neurosci..

[16] Luo AH, Tahsili-Fahadan P, Wise RA, Lupica CR, Aston-Jones G (2011). Linking context with reward: a functional circuit from hippocampal CA3 to ventral tegmental area. Science.

[17] Sjulson L, Peyrache A, Cumpelik A, Cassataro D, Buzsaki G (2018). Cocaine place conditioning strengthens location-specific hippocampal coupling to the nucleus accumbens. Neuron.

[18] Xia L, Nygard SK, Sobczak GG, Hourguettes NJ, Bruchas MR (2017). Dorsal-CA1 hippocampal neuronal ensembles encode nicotine-reward contextual associations. Cell Rep..

[19] Trouche S (2016). Recoding a cocaine-place memory engram to a neutral engram in the hippocampus. Nat. Neurosci..

[20] Vorel SR, Liu X, Hayes RJ, Spector JA, Gardner EL (2001). Relapse to cocaine-seeking after hippocampal theta burst stimulation. Science.

[21] Zhou Y (2019). A ventral CA1 to nucleus accumbens core engram circuit mediates conditioned place preference for cocaine. Nat. Neurosci..

[22] Ge F (2017). Glutamatergic projections from the entorhinal cortex to dorsal dentate gyrus mediate context-induced reinstatement of heroin seeking. Neuropsychopharmacology.

[23] McGlinchey EM, Aston-Jones G (2017). Dorsal hippocampus drives context-induced cocaine seeking via inputs to lateral septum. Neuropsychopharmacology.

[24] O’Keefe J, Dostrovsky J (1971). The hippocampus as a spatial map. Preliminary evidence from unit activity in the freely-moving rat. Brain Res..

[25] O’Keefe, J. & Nadel, L. *The Hippocampus as a Cognitive Map* (Clarendon Press, 1978).

[26] Muller RU, Kubie JL (1987). The effects of changes in the environment on the spatial firing of hippocampal complex-spike cells. J. Neurosci..

[27] Knierim JJ, Kudrimoti HS, McNaughton BL (1998). Interactions between idiothetic cues and external landmarks in the control of place cells and head direction cells. J. Neurophysiol..

[28] O’Keefe J, Conway DH (1978). Hippocampal place units in the freely moving rat: why they fire where they fire. Exp. Brain Res..

[29] Leutgeb S, Leutgeb JK, Treves A, Moser MB, Moser EI (2004). Distinct ensemble codes in hippocampal areas CA3 and CA1. Science.

[30] Leutgeb JK (2005). Progressive transformation of hippocampal neuronal representations in “morphed” environments. Neuron.

[31] Colgin LL (2010). Attractor-map versus autoassociation based attractor dynamics in the hippocampal network. J. Neurophysiol..

[32] Bostock E, Muller RU, Kubie JL (1991). Experience-dependent modifications of hippocampal place cell firing. Hippocampus.

[33] Frank LM, Brown EN, Wilson M (2000). Trajectory encoding in the hippocampus and entorhinal cortex. Neuron.

[34] Wood ER, Dudchenko PA, Robitsek RJ, Eichenbaum H (2000). Hippocampal neurons encode information about different types of memory episodes occurring in the same location. Neuron.

[35] Xu H, Baracskay P, O’Neill J, Csicsvari J (2019). Assembly responses of hippocampal CA1 place cells predict learned behavior in goal-directed spatial tasks on the radial eight-arm maze. Neuron.

[36] Dupret D, O’Neill J, Pleydell-Bouverie B, Csicsvari J (2010). The reorganization and reactivation of hippocampal maps predict spatial memory performance. Nat. Neurosci..

[37] Lever C, Wills T, Cacucci F, Burgess N, O’Keefe J (2002). Long-term plasticity in hippocampal place-cell representation of environmental geometry. Nature.

[38] Colgin LL, Moser EI, Moser MB (2008). Understanding memory through hippocampal remapping. Trends Neurosci..

[39] Leutgeb S (2005). Independent codes for spatial and episodic memory in hippocampal neuronal ensembles. Science.

[40] Mamad O (2017). Place field assembly distribution encodes preferred locations. PLoS Biol..

[41] Xiao Z, Lin K, Fellous JM (2020). Conjunctive reward-place coding properties of dorsal distal CA1 hippocampus cells. Biol. Cyber..

[42] Danielson NB (2016). Sublayer-specific coding dynamics during spatial navigation and learning in hippocampal area CA1. Neuron.

[43] Turi GF (2019). Vasoactive intestinal polypeptide-expressing interneurons in the hippocampus support goal-oriented spatial learning. Neuron.

[44] Kaufman AM, Geiller T, Losonczy A (2020). A role for the locus coeruleus in hippocampal CA1 place cell reorganization during spatial reward learning. Neuron.

[45] Zaremba JD (2017). Impaired hippocampal place cell dynamics in a mouse model of the 22q11.2 deletion. Nat. Neurosci..

[46] Hollup SA, Molden S, Donnett JG, Moser MB, Moser EI (2001). Accumulation of hippocampal place fields at the goal location in an annular watermaze task. J. Neurosci..

[47] Sanders, H., Wilson, M. A. & Gershman, S. J. Hippocampal remapping as hidden state inference. *eLife* **9**, 10.7554/eLife.51140 (2020). 10.7554/eLife.51140 PMC7282808 32515352

[48] Sun Y (2019). CA1-projecting subiculum neurons facilitate object-place learning. Nat. Neurosci..

[49] Ziv Y (2013). Long-term dynamics of CA1 hippocampal place codes. Nat. Neurosci..

[50] Cai DJ (2016). A shared neural ensemble links distinct contextual memories encoded close in time. Nature.

[51] Tzschentke TM (1998). Measuring reward with the conditioned place preference paradigm: a comprehensive review of drug effects, recent progress and new issues. Prog. Neurobiol..

[52] Tzschentke TM (2007). Measuring reward with the conditioned place preference (CPP) paradigm: update of the last decade. Addiction Biol..

[53] Sulzer D (2011). How addictive drugs disrupt presynaptic dopamine neurotransmission. Neuron.

[54] Zhou, P. et al. Efficient and accurate extraction of in vivo calcium signals from microendoscopic video data. *Elife* **7**, 10.7554/eLife.28728 (2018). 10.7554/eLife.28728 PMC5871355 29469809

[55] Gonzalez WG, Zhang H, Harutyunyan A, Lois C (2019). Persistence of neuronal representations through time and damage in the hippocampus. Science.

[56] Friedrich J, Zhou P, Paninski L (2017). Fast online deconvolution of calcium imaging data. PLoS Comput. Biol..

[57] Hardcastle K, Maheswaranathan N, Ganguli S, Giocomo LM (2017). A multiplexed, heterogeneous, and adaptive code for navigation in medial entorhinal cortex. Neuron.

[58] Ledergerber D (2021). Task-dependent mixed selectivity in the subiculum. Cell Rep..

[59] Mao D (2021). Spatial modulation of hippocampal activity in freely moving macaques. Neuron.

[60] Góis ZHTD, Tort ABL (2018). Characterizing speed cells in the rat hippocampus. Cell Rep..

[61] Muller RU, Bostock E, Taube JS, Kubie JL (1994). On the directional firing properties of hippocampal place cells. J. Neurosci..

[62] Gauthier JL, Tank DW (2018). A dedicated population for reward coding in the hippocampus. Neuron.

[63] Fyhn M, Molden S, Hollup S, Moser M-B, Moser EI (2002). Hippocampal neurons responding to first-time dislocation of a target object. Neuron.

[64] Marshel, J. H. et al. Cortical layer-specific critical dynamics triggering perception. *Science* **365**, 10.1126/science.aaw5202 (2019). 10.1126/science.aaw5202 PMC6711485 31320556

[65] Carrillo-Reid L, Han S, Yang W, Akrouh A, Yuste R (2019). Controlling visually guided behavior by holographic recalling of cortical ensembles. Cell.

[66] Robinson NTM (2020). Targeted activation of hippocampal place cells drives memory-guided spatial behavior. Cell.

[67] Sosa M, Giocomo LM (2021). Navigating for reward. Nat. Rev. Neurosci..

[68] Breese CR, Hampson RE, Deadwyler SA (1989). Hippocampal place cells: stereotypy and plasticity. J. Neurosci..

[69] Lee I, Griffin AL, Zilli EA, Eichenbaum H, Hasselmo ME (2006). Gradual translocation of spatial correlates of neuronal firing in the hippocampus toward prospective reward locations. Neuron.

[70] Kobayashi T, Tran AH, Nishijo H, Ono T, Matsumoto G (2003). Contribution of hippocampal place cell activity to learning and formation of goal-directed navigation in rats. Neuroscience.

[71] Hok V (2007). Goal-related activity in hippocampal place cells. J. Neurosci..

[72] Duvelle, É. et al. Insensitivity of place cells to the value of spatial goals in a two-choice flexible navigation task. *J. Neurosci.* 1578–1518, 10.1523/jneurosci.1578-18.2018 (2019). 10.1523/JNEUROSCI.1578-18.2018 PMC6435828 30696727

[73] Hölscher C, Jacob W, Mallot HA (2003). Reward modulates neuronal activity in the hippocampus of the rat. Behavioural Brain Res..

[74] Carmack, S. A., Koob, G. F. & Anagnostaras, S. G. *Learning and Memory in Addiction* 523–538, 10.1016/b978-0-12-809324-5.21101-2 (2017).

[75] Hyman SE, Malenka RC, Nestler EJ (2006). Neural mechanisms of addiction: the role of reward-related learning and memory. Annu. Rev. Neurosci..

[76] Skaggs WE, McNaughton BL (1998). Spatial firing properties of hippocampal CA1 populations in an environment containing two visually identical regions. J. Neurosci..

[77] Spiers HJ, Hayman RM, Jovalekic A, Marozzi E, Jeffery KJ (2015). Place field repetition and purely local remapping in a multicompartment environment. Cereb. Cortex.

[78] Derdikman D (2009). Fragmentation of grid cell maps in a multicompartment environment. Nat. Neurosci..

[79] Singer AC, Karlsson MP, Nathe AR, Carr MF, Frank LM (2010). Experience-dependent development of coordinated hippocampal spatial activity representing the similarity of related locations. J. Neurosci..

[80] Tabuchi E, Mulder AB, Wiener SI (2003). Reward value invariant place responses and reward site associated activity in hippocampal neurons of behaving rats. Hippocampus.

[81] Tryon VL (2017). Hippocampal neural activity reflects the economy of choices during goal-directed navigation. Hippocampus.

[82] Lee H, Ghim JW, Kim H, Lee D, Jung M (2012). Hippocampal neural correlates for values of experienced events. J. Neurosci..

[83] van der Meer, M. A. A., Ito, R., Lansink, C. S. & Pennartz, C. M. A. *Hippocampal Projections to the Ventral Striatum: from Spatial Memory to Motivated Behavior* 497–516, 10.1007/978-3-7091-1292-2_18 (2014).

[84] Lansink, C. S. & Pennartz, C. M. A. Associative reactivation of place–reward information in the hippocampal–ventral striatal circuitry. In *Analysis and Modeling of Coordinated Multi-neuronal Activity*. (ed. Tatsuno, M.) vol 12. 81–104 (Springer Series in Computational Neuroscience, 2015).

[85] Grosmark AD, Sparks FT, Davis MJ, Losonczy A (2021). Reactivation predicts the consolidation of unbiased long-term cognitive maps. Nat. Neurosci..

[86] Kempadoo KA, Mosharov EV, Choi SJ, Sulzer D, Kandel ER (2016). Dopamine release from the locus coeruleus to the dorsal hippocampus promotes spatial learning and memory. Proc. Natl Acad. Sci. USA.

[87] Takeuchi T (2016). Locus coeruleus and dopaminergic consolidation of everyday memory. Nature.

[88] McNamara CG, Tejero-Cantero Á, Trouche S, Campo-Urriza N, Dupret D (2014). Dopaminergic neurons promote hippocampal reactivation and spatial memory persistence. Nat. Neurosci..

[89] Rosen ZB, Cheung S, Siegelbaum SA (2015). Midbrain dopamine neurons bidirectionally regulate CA3-CA1 synaptic drive. Nat. Neurosci..

[90] Lisman JE, Grace AA (2005). The hippocampal-VTA loop: controlling the entry of information into long-term memory. Neuron.

[91] Teixeira CM (2018). Hippocampal 5-HT input regulates memory formation and schaffer collateral excitation. Neuron.

[92] Varga V (2009). Fast synaptic subcortical control of hippocampal circuits. Science.

[93] Brog JS, Salyapongse A, Deutch AY, Zahm DS (1993). The patterns of afferent innervation of the core and shell in the “Accumbens” part of the rat ventral striatum: Immunohistochemical detection of retrogradely transported fluoro-gold. J. Comp. Neurol..

[94] Trouche S (2019). A hippocampus-accumbens tripartite neuronal motif guides appetitive memory in space. Cell.

[95] Groenewegen HJ, der Zee EV-V, te Kortschot A, Witter MP (1987). Organization of the projections from the subiculum to the ventral striatum in the rat. A study using anterograde transport of Phaseolus vulgaris leucoagglutinin. Neuroscience.

[96] Ohara S (2018). Intrinsic projections of layer Vb neurons to layers Va, III, and II in the lateral and medial entorhinal cortex of the rat. Cell Rep..

[97] Sürmeli G (2015). Molecularly defined circuitry reveals input-output segregation in deep layers of the medial entorhinal cortex. Neuron.

[98] Boccara CN, Nardin M, Stella F, O’Neill J, Csicsvari J (2019). The entorhinal cognitive map is attracted to goals. Science.

[99] Butler WN, Hardcastle K, Giocomo LM (2019). Remembered reward locations restructure entorhinal spatial maps. Science.

[100] Steinmetz, N. A. et al. Aberrant cortical activity in multiple GCaMP6-expressing transgenic mouse lines. *eNeuro* **4**, 10.1523/ENEURO.0207-17.2017 (2017). 10.1523/ENEURO.0207-17.2017 PMC5604087 28932809

[101] Bardo MT, Bevins RA (2000). Conditioned place preference: what does it add to our preclinical understanding of drug reward?. Psychopharmacology.

[102] Cunningham CL, Ferree NK, Howard MA (2003). Apparatus bias and place conditioning with ethanol in mice. Psychopharmacology.

[103] Sambo, D. O. et al. The sigma-1 receptor modulates methamphetamine dysregulation of dopamine neurotransmission. *Nat. Commun.* **8**, 10.1038/s41467-017-02087-x (2017). 10.1038/s41467-017-02087-x PMC5738444 29263318

[104] Spiteri T, Le Pape G, Ågmo A (2000). What is learned during place preference conditioning? A comparison of food- and morphine-induced reward. Psychobiology.

[105] Lin R (2020). The raphe dopamine system controls the expression of incentive memory. Neuron.

[106] Gava GP (2021). Integrating new memories into the hippocampal network activity space. Nat. Neurosci..

[107] Zhu Y, Wienecke CFR, Nachtrab G, Chen X (2016). A thalamic input to the nucleus accumbens mediates opiate dependence. Nature.

[108] Pnevmatikakis EA, Giovannucci A (2017). NoRMCorre: an online algorithm for piecewise rigid motion correction of calcium imaging data. J. Neurosci. Methods.

[109] Pnevmatikakis EA (2016). Simultaneous denoising, deconvolution, and demixing of calcium imaging data. Neuron.

[110] Bolte S, Cordelieres FP (2006). A guided tour into subcellular colocalization analysis in light microscopy. J. Microsc.

[111] Skaggs WE, McNaughton BL, Wilson MA, Barnes CA (1996). Theta phase precession in hippocampal neuronal populations and the compression of temporal sequences. Hippocampus.

[112] Hussaini SA, Kempadoo KA, Thuault SJ, Siegelbaum SA, Kandel ER (2011). Increased size and stability of CA1 and CA3 place fields in HCN1 knockout mice. Neuron.

[113] Fenton AA (2008). Unmasking the CA1 ensemble place code by exposures to small and large environments: more place cells and multiple, irregularly arranged, and expanded place fields in the larger space. J. Neurosci..

[114] Zhang K, Ginzburg I, McNaughton BL, Sejnowski TJ (1998). Interpreting neuronal population activity by reconstruction: unified framework with application to hippocampal place cells. J. Neurophysiol..

[115] Tampuu A, Matiisen T, Olafsdottir HF, Barry C, Vicente R (2019). Efficient neural decoding of self-location with a deep recurrent network. PLoS Comput. Biol..

[116] Senbabaoglu Y, Michailidis G, Li JZ (2014). Critical limitations of consensus clustering in class discovery. Sci. Rep..

[117] Brunet JP, Tamayo P, Golub TR, Mesirov JP (2004). Metagenes and molecular pattern discovery using matrix factorization. Proc. Natl Acad. Sci. USA.

