# Supplementary material for: Neural circuit dynamics of drug-context associative learning in the mouse hippocampus
Source: Nat Commun. 2022 Nov 7;13:6721. doi: 10.1038/s41467-022-34114-x (PMC9640587; doi:10.1038/s41467-022-34114-x)
Supplement: Supplementary file 1 — Supplementary Information [file 41467_2022_34114_MOESM1_ESM.pdf]

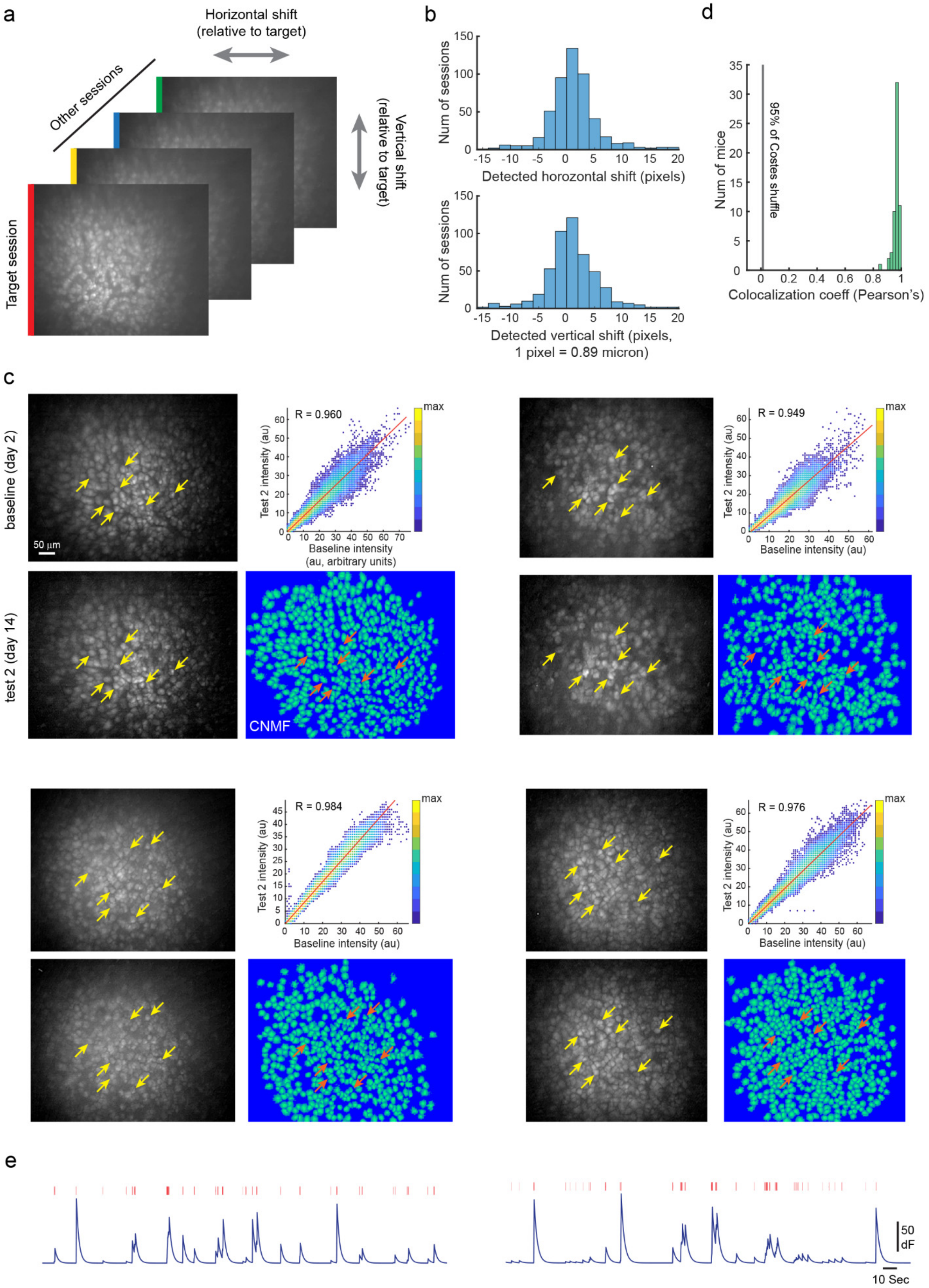

Supplementary Fig. 1, related to Fig. 1. Long-term single cell tracking of hippocampal CA1 neurons using a miniscope. a Methods for image alignment across different sessions. Images were motion corrected first for each session before the alignment. After the alignment, images from different sessions were concatenated together to extract neuronal activity. See also Supplementary Movies 1-3. **b** Detected horizontal (top) and vertical (bottom) shifts for the cross-session image alignment. Data was pooled across 501 aligned sessions from 54

mice. **c** Examples of single cell tracking from four representative mice. Each panel block is a mouse. For each block, maximum intensity projected images from baseline and test 2 sessions are shown on the left. Yellow arrows point to representative tracked neurons. Top right, cytofluorogram of colocalization analysis between baseline and test 2 images on the left. Fluorescence intensity for corresponding pixels of the two images are plotted. The figure is color coded for maximum (yellow) and minimum (blue) values with warmer colors indicate higher data point frequency. R value shows the Pearson's colocalization coefficient between the two images. Bottom right, CNMF-E spatial footprints of neurons extracted from the fully aligned and concatenated data. Orange arrows point to the extracted footprints corresponding to the neurons indicated by yellow arrows on the left. **d** Colocalization analyses between baseline and test 2 sessions for all mice (n = 54). Pearson's colocalization coefficient (green bar, 0.96 ± 0.0029, mean ± SEM) for each mouse and the 95th % of the Coste's shuffled correlation coefficient (grey line, 0.014 ± 0.0006) are shown in the histogram. **e** Representative denoised calcium signal traces (shown in dark blue) and deconvolved inferred spikes (shown as red bars) extracted from CNMF-E.

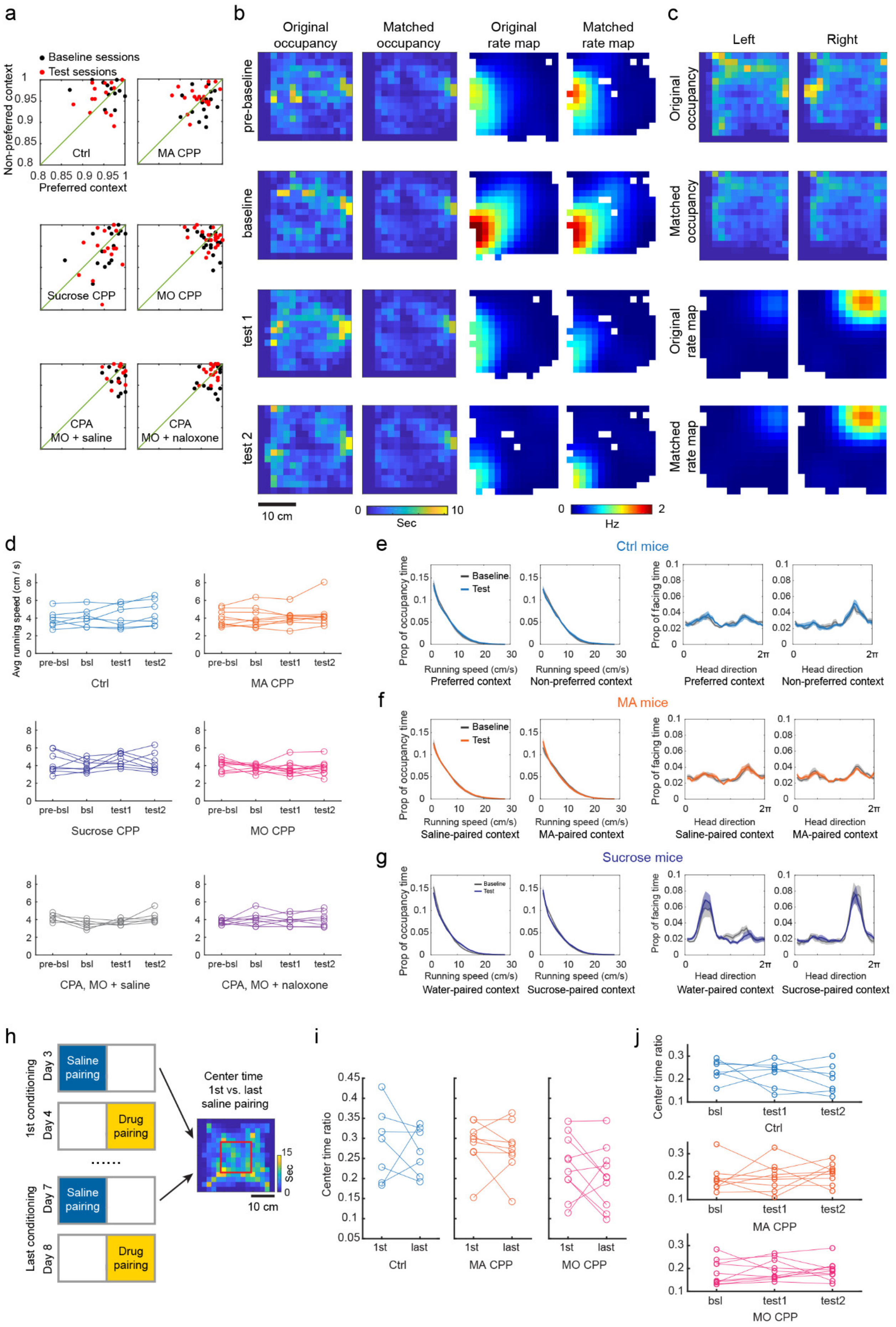

Supplementary Fig. 2, related to Fig. 2. Drug or natural reward conditioning did not change the animals’

spatial coverage, running speed, or time spent in the center of the arena. a Comparisons of animals' spatial coverage between the two CPP contexts from all the baseline and test sessions. Spatial coverage is quantified as the percentage of total spatial bins that an animal physically visited at least once within each session. Although drug/natural reward induced significant place preference in mice, spatial coverage was not significantly different between the two CPP contexts in either baseline or test sessions (preferred context vs. non-preferred context, all $p > 0.05$, n (from left to right) = 14, 18, 16, 20, 12, 16 sessions, respectively, two-tailed sign-rank test). **b** Performance of occupancy matching in the longitudinal domain. The first column shows the occupancy maps in the left CPP compartment for baseline and test sessions from a representative mouse. The occupancy map is color coded for minimum (blue) and maximum (yellow) values. The second column shows the same occupancy maps after the longitudinal matching/downsampling process. The third and fourth columns show the corresponding original vs. matched rate maps from a representative neuron. The rate map is color coded for minimum (blue) and maximum (red) values. **c** Similar to (b), but the performance of occupancy matching in the transverse domain (between the left and right CPP compartments). **d** Animals' average running speed from baseline and test sessions for all mice. Each line corresponds to an individual mouse. There were no differences in running speed in any given experiment (all $p > 0.05$, One-way ANOVA). **e - g** Speed and head direction sampling in Ctrl (e), MA (f), and Sucrose (g) mice in the preferred (saline) and non-preferred (reward-paired) contexts. Left two panels: mean (solid lines) ± SEM (shaded regions) of the proportion of time mice spent in each running speed bin. Black traces represent baseline sessions, colored traces represent test sessions. Right two panels: mean (solid lines) ± SEM (shaded regions) of the proportion of time mice spent in each head direction bin. Black traces represent baseline sessions, colored traces represent test sessions. There were no significant differences comparing between baseline and test sessions within each panel ($p > 0.05$ for all the panels, n = 7, 9, 8, respectively, two-tailed KS test). **h** Left, schematic shows the timeline for the 1st and last conditioning sessions. Right, a representative example for the occupancy time that the animal spent in the center of the arena in saline conditioning, denoted by the red box. The occupancy map is color coded for minimum (blue) and maximum (yellow) values. To compare occupancy across drug conditioning, time spent in the center of the arena was calculated for both the 1st and the last saline conditioning sessions, between which there were two drug-conditioning sessions. **i** Quantification of the proportion of time spent in the center of the arena in 1st vs. last saline conditioning (mean ± SEM, 1st vs. last, Ctrl: 0.29 ± 0.03 vs. 0.27 ± 0.02; MA: 0.29 ± 0.02 vs. 0.27 ± 0.02; MO: 0.23 ± 0.02 vs. 0.20 ± 0.02). There were no significant differences within the Ctrl, MA or MO group (Ctrl: $t(6) = 0.44$, $p = 0.67$; MA: $t(8) = 0.67$, $p = 0.52$; MO: $t(9) = 1.05$, $p = 0.32$, two-tailed paired t-test, n = 7, 9, 10, respectively). This suggests that exposure to the drug did not increase the anxiety level of the mice, which could be associated with drug withdrawal. **j** Quantification of the proportion of time spent in the center of the arena in baseline and test sessions (Ctrl: $F(2,20) = 0.72$, $p = 0.50$; MA: $F(2,26) = 0.3$, $p = 0.74$; MO: $F(2,29) = 0.14$, $p = 0.87$, One-way ANOVA). There was no change in the proportion of time the animals spend in the center of the arena between baseline and test sessions.

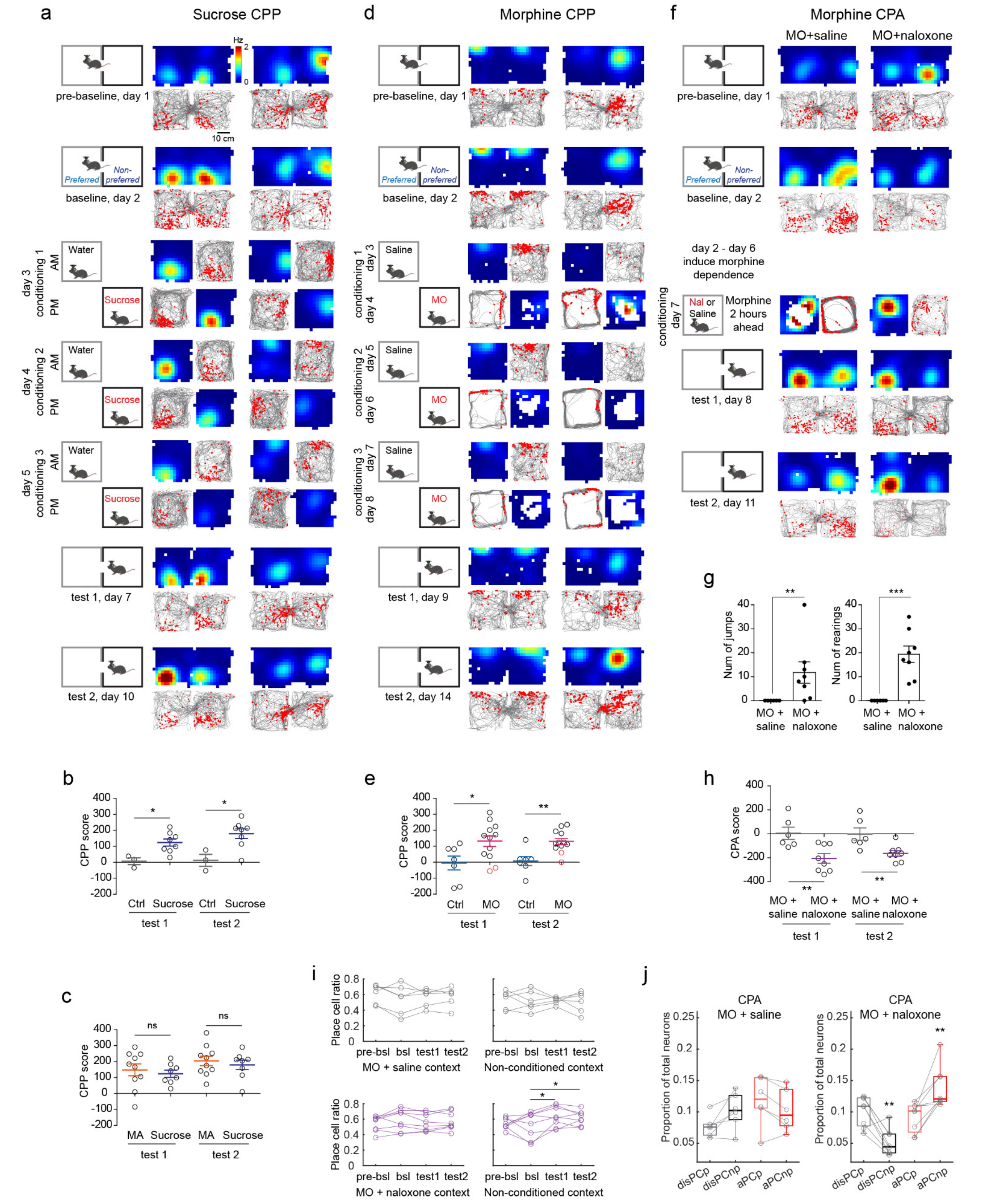

Supplementary Fig. 3, related to Fig. 2. Sucrose CPP, morphine CPP, and morphine CPA. a Left, schematic of the experimental design for sucrose CPP (see Methods). Right, longitudinally tracked place cell examples from two representative sucrose mice. Each column is a cell. For each cell, the spatial firing rate map (warmer colors indicate higher firing rates) and raster plot (red dots) on top of animals running trajectory (black traces)

are shown, organized according to the schematic on the left. **b** Behavioral CPP scores for sucrose mice. Sucrose mice showed a significant preference for the sucrose-paired context in both test sessions compared with Ctrl, which received water in all the conditioning sessions (mean ± SEM, test 1: Ctrl vs. Sucrose: 6.72 ± 21.44 vs. 123.7 ± 22.45 s, t(9) = 2.95, p = 0.016; test 2: Ctrl vs. Sucrose: 12.62 ± 37.35 vs. 179.4 ± 30.08 s, t(9) = 3.04, p = 0.014, n = 3 and 8, two-tailed unpaired t-test). **c** Comparisons of CPP scores between MA and sucrose mice. There was no significant difference between MA- vs. sucrose- induced place preference behaviors (test 1: t(16) = 0.52, p = 0.61; test 2: t(16) = 0.58, p = 0.57, n = 10 and 8, two-tailed unpaired t-test). Data are presented as mean ± SEM. **d** Similar to (a) but for morphine (MO) CPP. **e** Similar to (b) but for MO mice. MO mice showed a significant preference for the MO-paired context in both test sessions compared with Ctrl (mean ± SEM, test 1, Ctrl vs. MO: -5.62 ± 43.28 vs. 131.80 ± 33.48 s, t(17) = 2.50, p = 0.023; test 2, Ctrl vs. MO: 6.89 ± 28.67 vs. 130.10 ± 19.82 s; t(17) = 3.64, p = 0.0020, n = 7 and 12, two-tailed unpaired t-test). Red-colored data points indicate a neutral response to MO (defined as a negative CPP score). Data from these mice were excluded from further analyses. **f** To further validate that the observed place cell changes are due to the rewarding drug effects, we performed morphine conditioned place aversion (MO CPA) in a separate cohort of mice (MO+naloxone, n = 8 mice). Similar to (a) but for MO CPA. For rate map examples, one cell is from a MO+saline mouse and the other cell is from a MO+naloxone mouse as indicated. **g** Somatic withdrawal signs for MO CPA mice. Compared to MO+saline (control mice for MO CPA, n = 6 mice), naloxone precipitation (MO+naloxone) induced significant somatic withdrawal behaviors in the conditioning session. MO+naloxone mice showed a greater number of jumps and rearing during the 20-minute conditioning session, right after the injection of naloxone (** p = 0.0047, *** p = 6.6×10^{-4}, two-tailed rank-sum test). Data are presented as mean ± SEM. **h** Behavioral CPA scores for MO CPA mice. MO+naloxone mice showed a significant avoidance for the conditioning side in both test sessions compared with MO+saline mice (mean ± SEM, test 1: MO+saline vs. MO+naloxone: 4.27 ± 51.24 vs. -205.7 ± 39.78 s, t(12) = 3.29, p = 0.0064; test 2: MO+saline vs. MO+naloxone: -1.85 ± 39.78 vs. -163.4 ± 24.34 s, t(12) = 3.10, p = 0.0092, n = 6 and 8, two-tailed unpaired t-test). **i** In MO+naloxone mice, we found a significant increase in the number of place cells specifically in the non-conditioned context in test sessions compared to baseline. This effect was not seen in MO+saline mice. Top row, the ratio of place cells over the total number of neurons in MO + saline mice in the MO CPA experiments (MO + saline context: F(3,5) = 1.30, p = 0.31; non-conditioned context: F(3,5) = 0.14, p = 0.94, n = 6, repeated measures ANOVA). Bottom row, Same as the top row but for MO + naloxone mice in MO CPA (MO + naloxone context: F(3,7) = 1.11, p = 0.37; non-conditioned context: F(3,7) = 3.45, p = 0.035, n = 8, repeated measures ANOVA). The number of place cells in the non-conditioned context increased in the test sessions (mean ± SEM, test 1: 0.63 ± 0.05, test 2: 0.62 ± 0.03) compared to the baseline (0.53 ± 0.06) (bsl vs. test 1: t(7) = 3.16, p = 0.016; bsl vs. test 2: t(7) = 2.88, p = 0.024, n = 8, two-tailed paired t-test). Note, MO + saline/naloxone context is equivalent to the preferred context and non-conditioned context is equivalent to the non-preferred/drug-paired context in the CPP experiments. pre-bsl: pre-baseline, bsl: baseline. **j** In MO+naloxone mice, the place cell increase was due to a smaller proportion of disPCnp and a greater proportion of aPCnp. Left, the proportion of defined functional cell types for MO + saline mice in MO CPA (mean ± SEM, disPCp vs. disPCnp: 0.077 ± 0.007 vs. 0.10 ± 0.01, t(5) = -2.56, p = 0.051; aPCp vs. aPCnp: 0.12 ± 0.02 vs. 0.10 ± 0.01; t(5) = -2.27, p = 0.07, n = 6, two-tailed paired t-test). Right, same as the left panel but for MO + naloxone mice in MO CPA (mean ± SEM, disPCp vs. disPCnp: 0.10 ± 0.008 vs. 0.051 ± 0.007, t(7) = 4.55, p = 0.0026; aPCp vs. aPCnp: 0.092 ± 0.008 vs. 0.14 ± 0.01; t(7) = 4.01, p = 0.0051, n = 8, two-tailed paired t-test). The effect of MO CPA on place cells was thus the opposite of the changes in place cells observed in MA or MO CPP, suggesting that the place cell changes observed in drug CPP cannot be ascribed to drug withdrawal. For each box plot, the center indicates median, the box indicates 25th and 75th percentiles. The whiskers extend to the most extreme data points without outliers. Circles denote data points for each mouse.

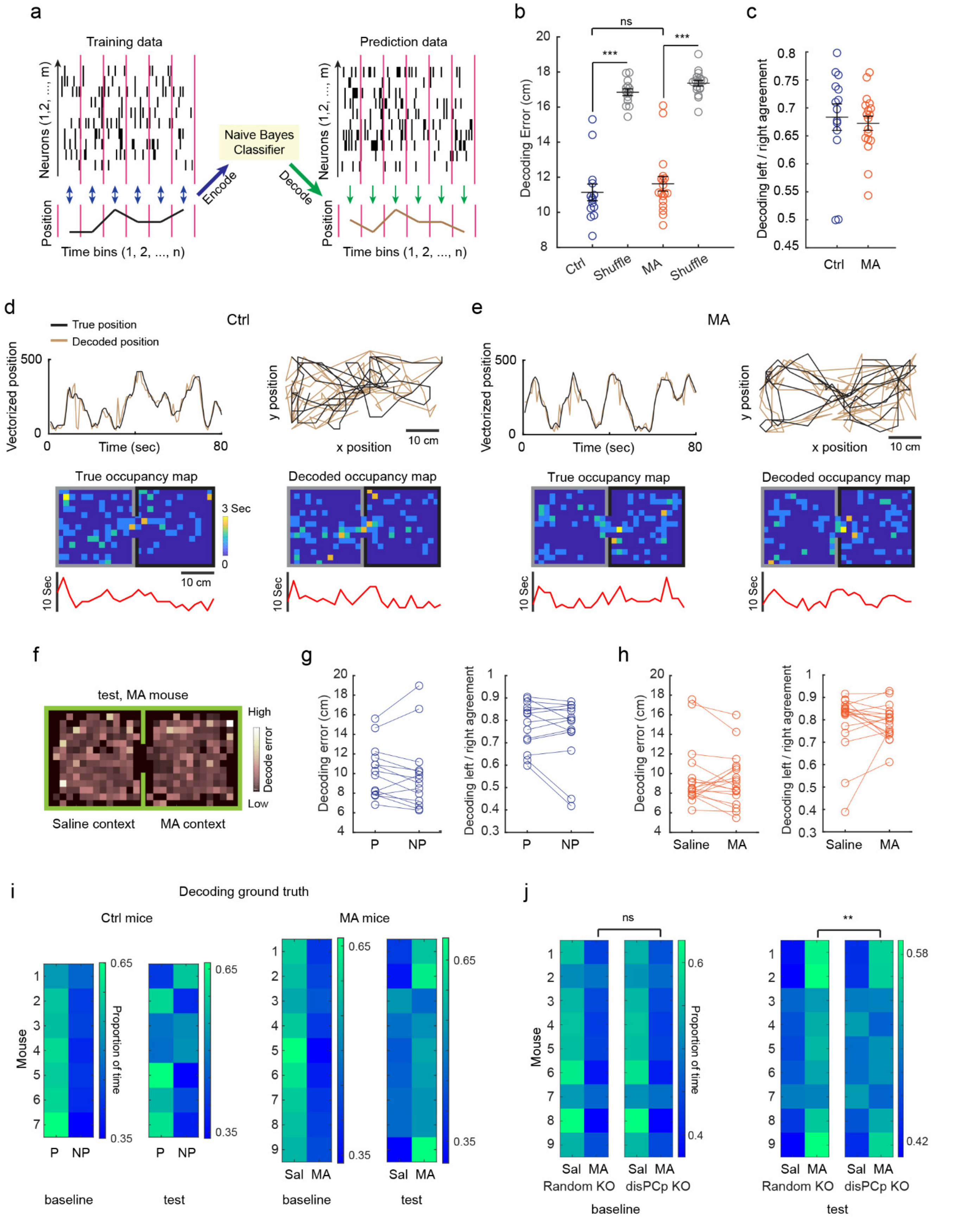

Supplementary Fig. 4, related to Fig. 3 and 4. MA-induced place cell activity change does not impair spatial decoding performance. a Schematic illustration of training (encoding) and prediction (decoding) processes for the naïve Bayes classifier. Neural and behavioral data were binned at 800 ms. The black trace indicates the true position of the animal, while the brown trace indicates the predicted/decoded position. **b - c**

Decoding performance for Ctrl mice, MA mice and shuffled conditions. The decoder was trained using baseline data in which the number of neurons was randomly down-sampled such that a comparable number was used across different animals. Predictions were then made using data from test sessions with the same down-sampling approach. We performed this down-sampling 50 times for each mouse and the final results were averaged from these 50 iterations. While decoding errors were significantly better than the shuffle for both groups (panel b, mean ± SEM, Ctrl vs. shuffle: 11.14 ± 0.47 vs. 16.85 ± 0.19 cm, $p = 1.22 \times 10^{-4}$; MA vs. shuffle: 11.63 ± 0.42 vs. 17.36 ± 0.16 cm, Z = -3.72, $p = 1.96 \times 10^{-4}$, two-tailed sign-rank test, n = 14 and 18 sessions. Note: two test sessions for each animal), there were no differences in either the decoding errors (panel b, Ctrl vs. MA: Z = -1.42, p = 0.15, two-tailed rank sum test) or decoding agreements (panel c, Ctrl 0.68 ± 0.02 vs. MA 0.67 ± 0.01, Z = 1.04, p = 0.30, two-tailed rank sum test) between Ctrl and MA mice. **d** Top, an example of the true vs. decoded spatial position of a Ctrl mouse in 1D (left) and 2D (right) over an 80-second period. Bottom, example occupancy map of the true vs. decoded spatial position for the same Ctrl mouse and data shown on top. The red trace at the bottom shows summed time for each column of spatial bins in the corresponding occupancy map. **e** Organized as in (d), for a representative MA mouse. **f** A 2D visualization of the decoding error in each spatial bin of the CPP environment from a test session of an MA mouse. Brighter colors indicate higher decoding errors. **g** Comparisons of decoding error (mean ± SEM, preferred context (P) : 10.19 ± 0.71, non-preferred context (NP): 9.9 ± 0.99 cm, p = 0.50, two-tailed sign-rank test, n = 14) and decoding agreement (P: 0.78 ± 0.03, NP: 0.75 ± 0.04, p = 0.81, two-tailed sign-rank test, n = 14) across the two CPP contexts in test sessions of Ctrl mice. The decoder was trained using baseline data and predictions were made using test session data. Each animal's occupancy was matched between the two compartments for both the training and prediction. We performed this occupancy matching 50 times for each mouse and the final results were averaged from these 50 iterations. As the comparisons were within subjects, the analyses were performed without down-sampling the number of neurons. **h** Organized as in (g), comparisons of decoding error (mean ± SEM, saline-paired context: 9.56 ± 0.73, MA-paired context: 9.33 ± 0.64 cm, Z = 0.63, p = 0.53, two-tailed sign-rank test, n = 18) and decoding agreement (saline-paired context: 0.79 ± 0.03, MA-paired context: 0.79 ± 0.02, Z = 0.46, p = 0.65, two-tailed sign-rank test, n = 18) in test sessions of MA mice. **i** Related to Fig. 4c-d, showing the ground truth for the reconstructed CPP time in Ctrl (left) and MA (right) mice. The proportion of total time on the non-preferred [NP] context in Ctrl mice is 0.41 ± 0.01 (baseline, mean ± SEM) and 0.45 ± 0.03 (test), n = 7. The proportion of total time on the drug-paired [MA] context in MA mice is 0.41 ± 0.01 (baseline) and 0.57 ± 0.02 (test), n = 9. **j** Same analysis in MA mice as shown in Fig. 4d, but with a downsampled (matched to Ctrl level) number of disPCp knocked out in the analysis. This analysis showed the same results as in Fig. 4d, regardless the number of disPCp were used (Left, baseline analyses, p = 0.65; Right, test analyses, p = 0.0039, two-tailed sign-rank test, n = 9).

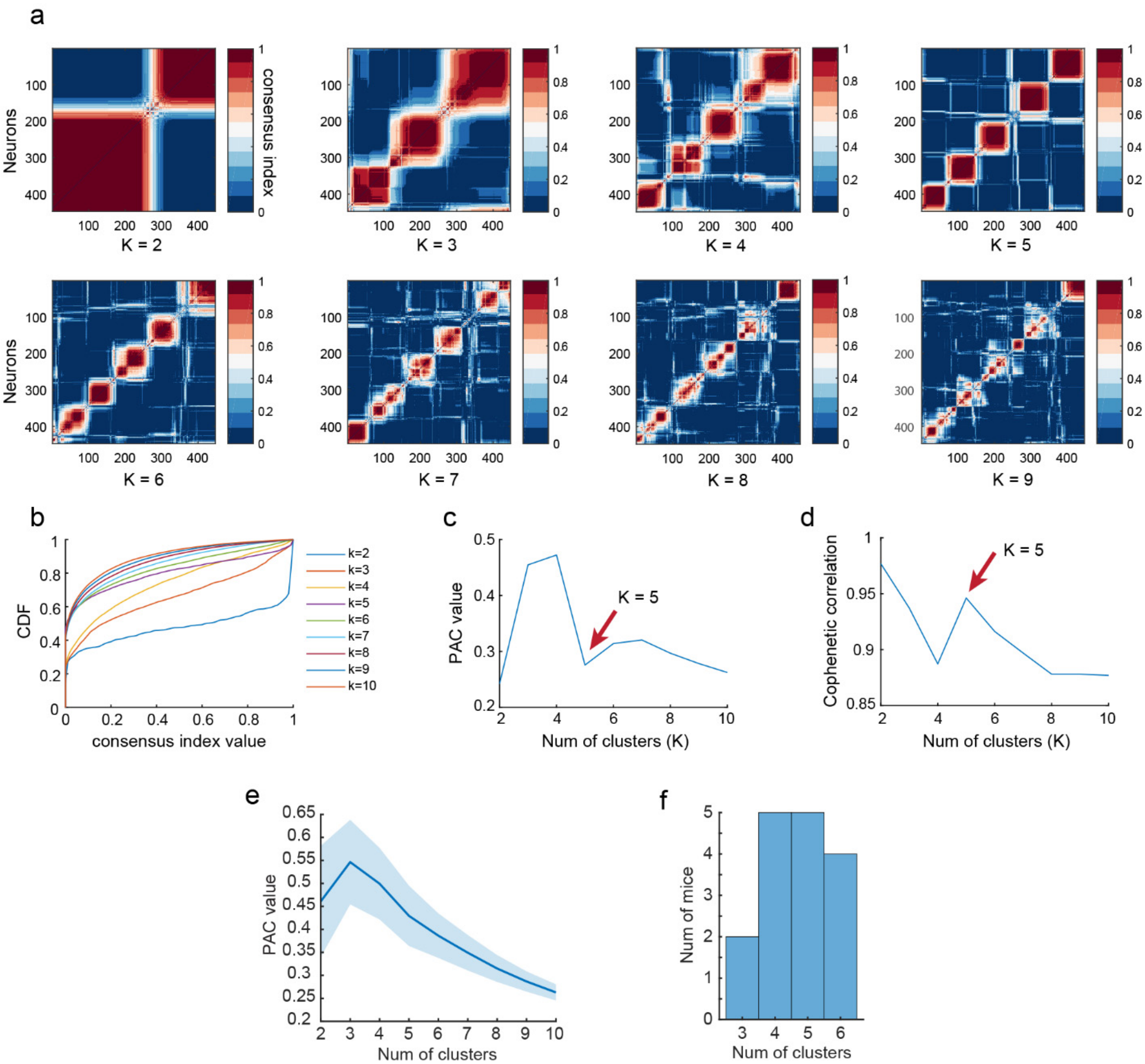

Supplementary Fig. 5, related to Fig. 5. Determining the optimal number of temporal clusters using a consensus k-means method. a Consensus matrices obtained at given numbers of clusters (K) using data from the baseline session of an MA mouse. The matrices are colored code for high (red) and low (blue) consensus index values. The consensus matrix was computed based on k-means clustering using randomly sub-sampled data with 100 iterations. Neuron pairs which showed the same cluster assignment in many of the iterations will have a high consensus index value. On the other hand, neuron pairs which rarely show the same cluster assignment will have a low consensus value. The matrices shown in the figure were re-organized using a hierarchical clustering method with complete linkage. **b** An example cumulative distribution function (CDF) plot of the consensus index values, at different numbers of clusters, obtained from the same data set in (a). A consensus index value CDF with perfect clustering should follow a Bernoulli distribution and show a flattened curve in the middle. **c** An example mouse in which we used the Proportion of Ambiguous Clustering (PAC) value to determine the optimal number of clusters on the baseline data. PAC was calculated using the proportion of data points with a consensus index value between 0.1 – 0.9 on the CDF plot (see panel b). The optimal number of clusters is indicated by the local minima (red arrow). **d** An example mouse in which we used the cophenetic correlation to determine the optimal number of clusters in the baseline data, which is indicated by the local maxima (red arrow). **e** Averaged PAC value for the baseline data of Ctrl and MA mice (n = 16 mice) at given numbers of clusters. Note the elbow shape is at K = 5. Data are plotted with mean (dark blue line) ± SEM (light blue shading). **f** Histogram showing the number of mice (both Ctrl and MA) with their optimal number of clusters (n = 16 mice), as determined by the cophenetic correlation. Together, given the data plotted in (e) and (f), the optimal number of clusters K = 5 was used for all analyses.

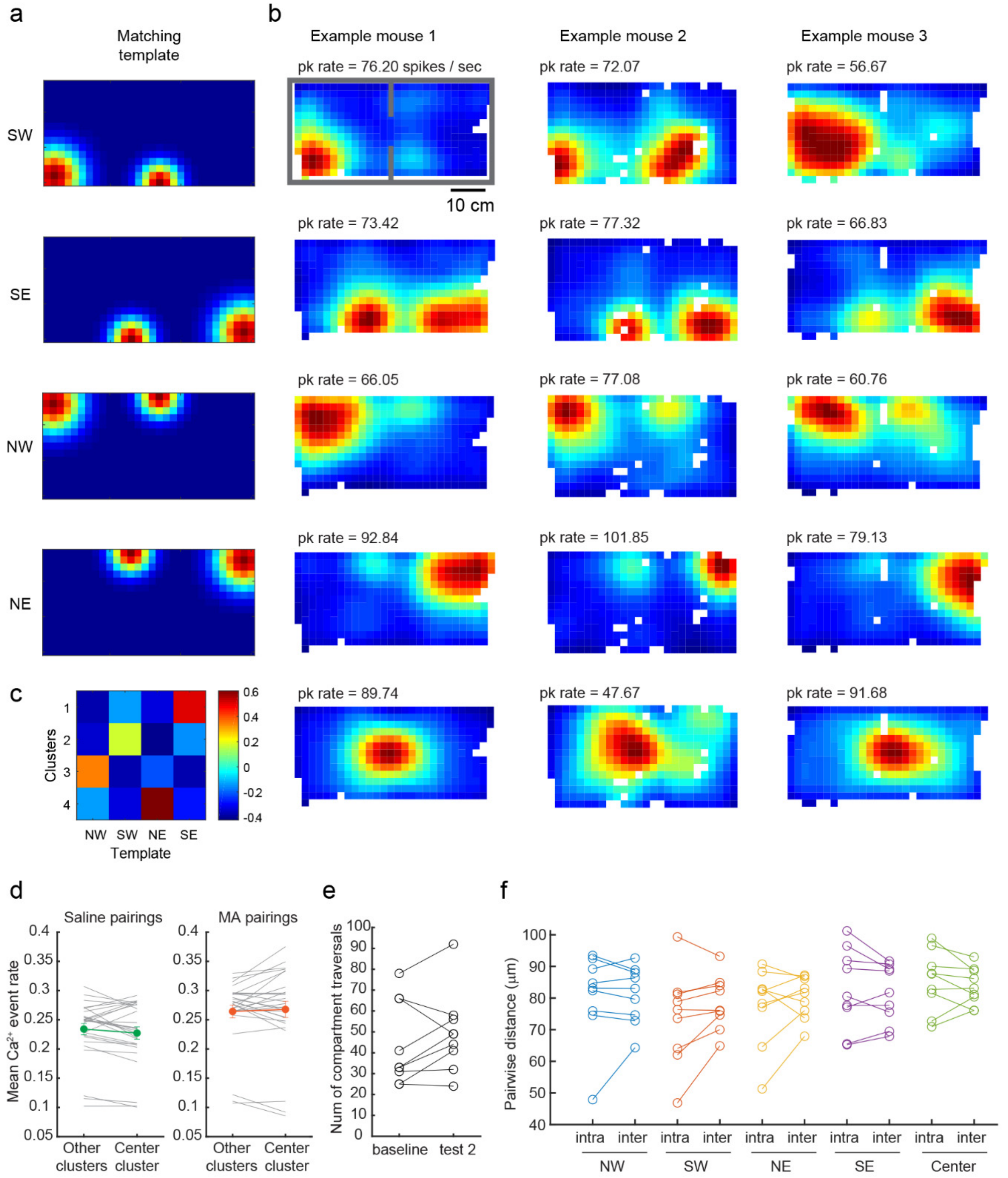

Supplementary Fig. 6, related to Fig. 5. A template matching method to sort temporal clusters across animals using their spatial firing patterns. a Gaussian spatial firing pattern templates used for matching (see methods). SW, southwest; SE, southeast; NW, northwest; NE, northeast. Templates are color coded for minimum (blue) and maximum (red) values to mimic the spatial firing pattern of the ensemble activity observed in temporal clusters (shown in b). In brief, the matching process is to compute a Pearson's correlation coefficient between an ensemble spatial rate map from a given temporal cluster (i.e., a summed rate map of all neurons from the cluster, shown in b) and the Gaussian templates, then find the template with the highest correlation. To avoid aberrant matching performance, the 'center' group, which can be unambiguously identified, was held out during this matching process. **b** Ensemble spatial rate maps for the five temporal clusters from three example mice. The rate maps are color coded for minimum (blue) and maximum (red) values with peak Ca^{2+} event rates indicated on the top left and organized according to the best matched template in (a). **c** The correlation matrix resulting from the template matching process from an example mouse. Each cluster can be unambiguously assigned to a template based on the highest correlation value in each column. **d** The activity of neurons from center vs. other clusters in saline or MA conditioning sessions of MA mice ($Z = 1.54$, $p = 0.12$ and $Z = -0.41$, $p =$

0.68, two-tailed sign-rank test, n = 27 conditioning sessions from 9 mice). Gray lines represent individual conditioning sessions, colored lines indicate the mean ± SEM (left, 0.23 ± 0.01 vs. 0.23 ± 0.01; right, 0.26 ± 0.01 vs. 0.27 ± 0.01). **e** The number of inter-compartment traversals between the baseline and test 2 in MA mice (mean ± SEM, 44.22 ± 6.73 vs. 49.44 ± 6.43, t(8) = -1.29, p = 0.23, two-tailed paired t-test, n = 9 mice). **f** There was no difference between the pairwise intra- vs. inter-cluster anatomical distances for any disPCp cluster in MA mice (intra vs. inter: mean ± SEM (μm), NW: 80.43 ± 4.62 vs. 81.17 ± 3.04, t(8) = -0.34, p = 0.74; SW: 73.79 ± 4.95 vs. 78.43 ± 2.85, t(8) = -1.97, p = 0.08; NE: 77.55 ± 4.13 vs. 80.16 ± 2.12, t(8) = -0.95, p = 0.37; SE: 82.77 ± 4.29 vs. 81.39 ± 3.06, t(8) = 0.83, p = 0.43; Center: 85.42 ± 3.19 vs. 83.76 ± 1.98, t(8) = 0.88, p = 0.40; two-tailed paired t-test, n = 9).

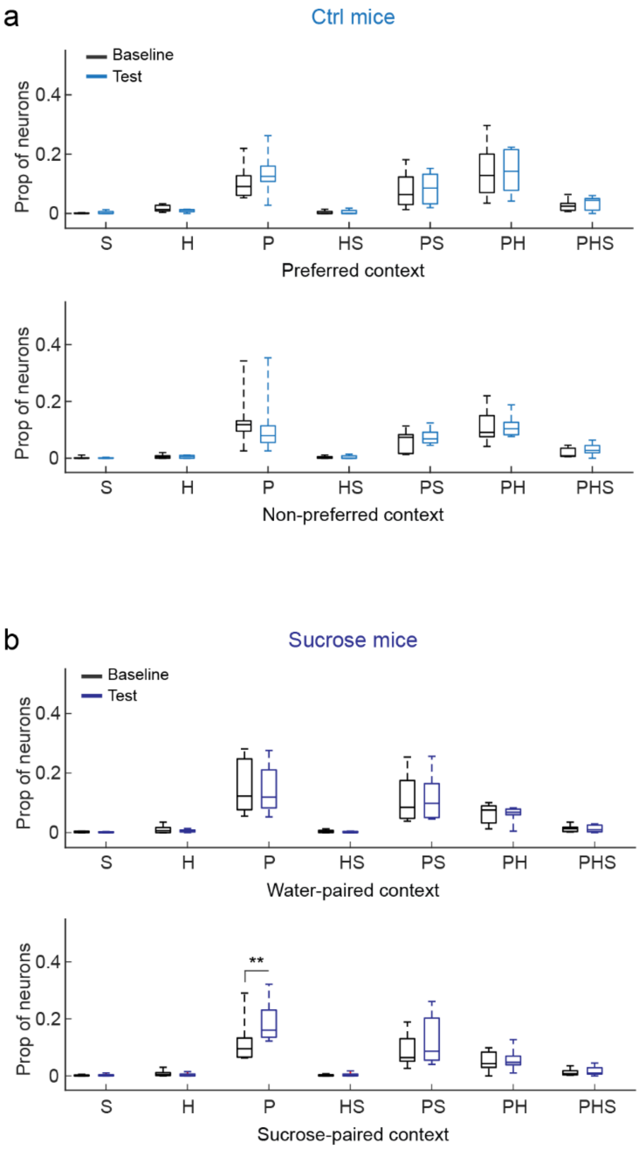

Supplementary Fig. 7, related to Fig. 6. LN results for Ctrl and Sucrose mice. a Proportion of total neurons for each cell type (P = position, S = speed, H = head direction) in baseline and test sessions of Ctrl mice. No significant changes were observed between baseline and test sessions. **b** Same as (d) but for sucrose mice. There was a significant increase in the number of P encoding neurons in test sessions compared to baseline, specifically in the sucrose-paired context (equivalent to drug-paired context in MA mice) (** $p = 0.0078$, two-tailed sign-rank test, $n = 9$ mice). For box plots throughout the figure, the center indicates median, the box indicates 25th and 75th percentiles. The whiskers extend to the most extreme data points without outliers.
