# Supplementary material for: Neural circuit dynamics of drug-context associative learning in the mouse hippocampus
Source: Nat Commun. 2022 Nov 7;13:6721. doi: 10.1038/s41467-022-34114-x (PMC9640587; doi:10.1038/s41467-022-34114-x)
Supplement: Supplementary file 2 — Description of Additional Supplementary Files [file 41467_2022_34114_MOESM2_ESM.pdf]

Description of Additional Supplementary Files

File Name: Supplementary Movie 1
Description: the performance of motion correction on a raw video clip of an example mouse.

File Name: Supplementary Movie 2
Description: the performance of cross-session alignment on the maximum projected images of baseline and test sessions from an example mouse.

File Name: Supplementary Movie 3
Description: an example tracked neurons (white circle) through baseline to test sessions.
