# Supplementary material for: Neural circuit dynamics of drug-context associative learning in the mouse hippocampus
Source: Nat Commun. 2022 Nov 7;13:6721. doi: 10.1038/s41467-022-34114-x (PMC9640587; doi:10.1038/s41467-022-34114-x)
Supplement: Supplementary file 6 — Reporting Summary [file 41467_2022_34114_MOESM6_ESM.pdf]

nature portfolio

Corresponding author(s): Lisa M Giocomo, Yanjun Sun

Last updated by author(s): Sept 26, 2022

Reporting Summary

Nature Portfolio wishes to improve the reproducibility of the work that we publish. This form provides structure for consistency and transparency in reporting. For further information on Nature Portfolio policies, see our Editorial Policies and the Editorial Policy Checklist.

Statistics

For all statistical analyses, confirm that the following items are present in the figure legend, table legend, main text, or Methods section.

n/a	Confirmed	
☐	☒	The exact sample size (*n*) for each experimental group/condition, given as a discrete number and unit of measurement
☐	☒	A statement on whether measurements were taken from distinct samples or whether the same sample was measured repeatedly
☐	☒	The statistical test(s) used AND whether they are one- or two-sided<br>*Only common tests should be described solely by name; describe more complex techniques in the Methods section.*
☐	☒	A description of all covariates tested
☐	☒	A description of any assumptions or corrections, such as tests of normality and adjustment for multiple comparisons
☐	☒	A full description of the statistical parameters including central tendency (e.g. means) or other basic estimates (e.g. regression coefficient) AND variation (e.g. standard deviation) or associated estimates of uncertainty (e.g. confidence intervals)
☐	☒	For null hypothesis testing, the test statistic (e.g. *F*, *t*, *r*) with confidence intervals, effect sizes, degrees of freedom and *P* value noted<br>*Give P values as exact values whenever suitable.*
☐	☒	For Bayesian analysis, information on the choice of priors and Markov chain Monte Carlo settings
☒	☐	For hierarchical and complex designs, identification of the appropriate level for tests and full reporting of outcomes
☐	☒	Estimates of effect sizes (e.g. Cohen's *d*, Pearson's *r*), indicating how they were calculated

Our web collection on statistics for biologists contains articles on many of the points above.

Software and code

Policy information about availability of computer code

Data collection	Ca++ Imaging data were acquired using custom-developed and validated software (Compiled DAQ Software OLD, https://github.com/daharoni/Miniscope_DAQ_Software)
Data analysis	Ca++ imaging data were preprocessed and analyzed using MATLAB based packages and scripts. MATLAB packages that were used for the manuscript include NoRMCorre v1.0.0 (https://github.com/flatironinstitute/NoRMCorre) for image motion correction, image-registration (github.com/fordanic/image-registration) for image alignment across days, CNMF-E (https://github.com/zhoupc/CNMF_E) for Ca++ signal extraction, and OASIS (https://github.com/zhoupc/OASIS_matlab) for Ca++ signal deconvolution. MATLAB 2017b and 2020a was used to perform statistical analyses. Custom MATLAB code generated in this study is available at: https://github.com/yanjuns/Sun_Giocomo_2022_NComms

For manuscripts utilizing custom algorithms or software that are central to the research but not yet described in published literature, software must be made available to editors and reviewers. We strongly encourage code deposition in a community repository (e.g. GitHub). See the Nature Portfolio guidelines for submitting code & software for further information.

Data

Policy information about availability of data

All manuscripts must include a data availability statement. This statement should provide the following information, where applicable:

- Accession codes, unique identifiers, or web links for publicly available datasets
- A description of any restrictions on data availability
- For clinical datasets or third party data, please ensure that the statement adheres to our policy

The datasets generated in the current study are available on Mendeley Data: https://doi.org/10.17632/p8gh7wk9z3.1

Field-specific reporting

Please select the one below that is the best fit for your research. If you are not sure, read the appropriate sections before making your selection.

☒ Life sciences ☐ Behavioural & social sciences ☐ Ecological, evolutionary & environmental sciences

For a reference copy of the document with all sections, see nature.com/documents/nr-reporting-summary-flat.pdf

Life sciences study design

All studies must disclose on these points even when the disclosure is negative.

Sample size	Depending on the analysis in question, sample size was determined by the number of mice or number of recording sessions. Sample size and the type of data used to determine it are reported appropriately in the manuscript. The sample sizes were not calculated by power analyses; however, our sample sizes are comparable or higher than those previously used in the literature and our results are consistent across animals (Sjulson et al., 2018, Xia et al., 2017, Trouche et al., 2016).
Data exclusions	Prior to the CPP experiments, we defined an exclusion threshold, in which we would exclude any mouse that spent more than 75% of their total time in one compartment in the baseline session; however, no mice reached this threshold thus no mice were excluded.
Replication	Up to six mice were used as a cohort for each batch of experiments. Key experiments (Ctrl, MA, MO) were repeated with at least two different cohorts of mice and the results are reproducible.
Randomization	Animals were randomly assigned to experimental groups.
Blinding	Experimenters were not blinded to experimental groups. Experimental groups received drug treatments which produce obvious behavior effects, thus by witnessing the animal undergoing the experiment, any blinding that would have been attempted would be compromised. It would be impossible to blind the experimenter to the experimental conditions while still being physically present during the experiment. Presence during the experiment is required by the ethics committee overseeing the experiment.

Reporting for specific materials, systems and methods

We require information from authors about some types of materials, experimental systems and methods used in many studies. Here, indicate whether each material, system or method listed is relevant to your study. If you are not sure if a list item applies to your research, read the appropriate section before selecting a response.

Materials & experimental systems

n/a	Involved in the study	
☒	☐	Antibodies
☒	☐	Eukaryotic cell lines
☒	☐	Palaeontology and archaeology
☐	☒	Animals and other organisms
☒	☐	Human research participants
☒	☐	Clinical data
☒	☐	Dual use research of concern

Methods

n/a	Involved in the study	
☒	☐	ChIP-seq
☒	☐	Flow cytometry
☒	☐	MRI-based neuroimaging

Animals and other organisms

Policy information about studies involving animals; ARRIVE guidelines recommended for reporting animal research

Laboratory animals	Ai94; CaMKIIa-tTA; CaMKIIa-Cre triple transgenic mice were used (Jackson Laboratory stock #024115, 8 -12 weeks old). A total of 54 mice were used in this study (30 males and 24 females). Each group contains both sex and the analyses were carried out by combining the data from both sex.
Wild animals	No wild animals were used in this study
Field-collected samples	No field collected samples were used in this study.
Ethics oversight	All procedures were approved by the Institutional Animal Care and Use Committee at Stanford University School of Medicine.

Note that full information on the approval of the study protocol must also be provided in the manuscript.
